# Development assistance for health and the Middle East and North Africa

**DOI:** 10.1186/s12992-020-0545-z

**Published:** 2020-02-04

**Authors:** Yingxi Zhao, Angela E. Micah, Stephen Gloyd, Joseph L. Dieleman

**Affiliations:** 1 Department of Global Health, University of Washington, Seattle, WA 98195 USA; 2 Institute for Health Metrics and Evaluation, University of Washington, Seattle, WA USA

**Keywords:** Development assistance for health, Global health, Middle East, Arab

## Abstract

**Background:**

Donor countries in the Middle East and North Africa (MENA) including Saudi Arabia, Kuwait and United Arab Emirates (UAE) have been among the largest donors in the world. However, little is known about their contributions for health. In this study, we addressed this gap by estimating the amount of development assistance for health (DAH) contributed by MENA country donors from 2000 to 2017.

**Methods:**

We tracked DAH provided and received by the MENA region leveraging publicly available development assistance data in the Development Assistance Committee (DAC) database of the Organisation for Economic Co-operation and Development (OECD), government agency reports and financial statements from key international development agencies. We generated estimates of DAH provided by the three largest donor countries in the MENA region (UAE, Kuwait, Saudi Arabia) and compared contributions to their relative gross domestic product (GDP) and government spending; We captured DAH contributions by other MENA country governments (Egypt, Iran, Qatar, Turkey, etc.) disbursed through multilateral agencies. Additionally, we compared DAH contributed from and provided to the MENA region.

**Results:**

In 2017, DAH contributed by the MENA region reached $514.8 million. While UAE ($220.1 million, 43.2%), Saudi Arabia ($177.3 million, 34.8%) and Kuwait ($59.8 million, 11.6%) as sources contributed the majority of DAH in 2017, 58.5% of total DAH from MENA was disbursed through their bilateral agencies, 12.0% through the World Health Organization (WHO) and 3.3% through other United Nations agencies. 44.8% of DAH contributions from MENA was directed to health system strengthening/sector-wide approaches. Relative to their GDP and government spending, DAH level fluctuated across 2000 to 2017 but UAE and Saudi Arabia indicated increasing trends. While considering all MENA countries as recipients, only 10.5% of DAH received by MENA countries were from MENA donors in 2017.

**Conclusion:**

MENA country donors especially UAE, Saudi Arabia and Kuwait have been providing substantial amount of DAH, channeled through their bilateral agencies, WHO and other multilateral agencies, with a prioritized focus on health system strengthening. DAH from the MENA region has been increasing for the past decade and could lend itself to important contributions for the region and the globe.

## Background

Development assistance for health (DAH), defined as the financial and in-kind contributions transferred through major development agencies to low- and middle-income countries for maintaining or improving health, has held steady in annual growth rate since 2011. According to the Institute for Health Metrics and Evaluation’s (IHME) latest Financing Global Health 2018 report, DAH has experienced a 0.3% drop in the annual growth rate over the most recent 5 years (2013–2018) [1]. Political uncertainties, changing commitment from traditional donors like the United States and the United Kingdom [2] and the large financial gap needed to achieve the Sustainable Development Goals [3], suggests that other sources may be critical to growing funding in the future.

**Fig. 1:**
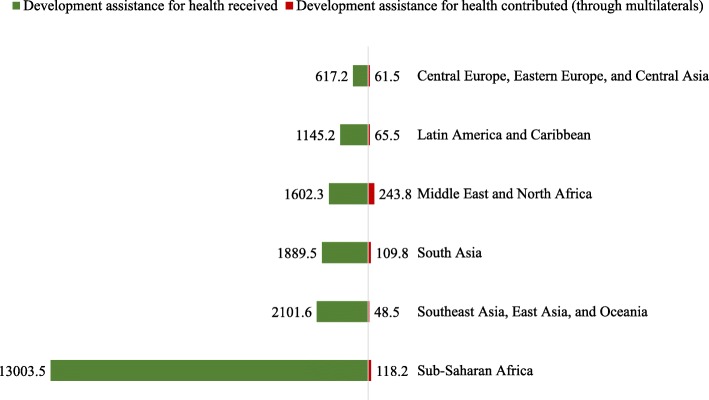
Development assistance for health received and contributed by Global Burden of Diseases Super-region, 2015–2017. Note: Values are 2015–2017 average values expressed in millions of 2018 US dollars. Development assistance provided by Global Burden of Diseases’ “high-income countries” super-region, development assistance received by global initiatives and unallocable region are not shown in the figure. Development assistance for health contributed through multilaterals include contribution through the World Health Organization, United Nations Children’s Fund, United Nations Population Fund, Pan American Health Organization, the World Bank, Gavi, the Global Fund, Unitaid, Coalition for Epidemic Preparedness Innovation and non-governmental organizations

Besides the traditional donor countries, who are usually members of the Organisation for Economic Co-operation and Development’s (OECD) Development Assistance Committee (DAC) and usually high-income countries, several other middle-income countries have gradually emerged in the global health financing arena. Brazil, Russia, India, China and South Africa, commonly referred to as the BRICS countries, are making a number of important commitments towards global health through providing development aid under the “South-south cooperation” regime and have also emphasized international cooperation including technology transfer to developing countries in the BRICS health ministers’ meetings [4]. Furthermore, Arab donors, like Saudi Arabia, United Arab Emirates and Kuwait have been providing development aid for decades. More recently, countries like Turkey have also started disbursing development assistance bilaterally. Most of these non-traditional donors do not report their bilateral aid to OECD DAC, which limits our understanding of their financial contributions, however estimates from IHME’s Financing Global Health 2018 analysis (Fig. 1) indicated that DAH contributed through multilateral agencies by different region (high-income countries excluded) varied between $61.5 million (Central Europe, Eastern Europe, and Central Asia) to $243.8 million (Middle East and North Africa).

The Middle East and North Africa (MENA) is a diverse region that has been in turmoil since the Arab spring, with Syria, Libya, Yemen and other countries experiencing ongoing civil war, and Jordan, Lebanon among others in the midst of the biggest refugee crisis since World War II [5]. The unstable condition and geopolitical tension have led to the influx of humanitarian aid in several MENA countries. In 2016, countries in MENA received over a third of total OECD DAC’s humanitarian flows [6], and specifically for health, an annual average of $1602.3 million from 2015 to 2017 (Fig. 1).

Meanwhile, the region holds a significant share of the world’s energy resources and also some of the most substantial aid contributors. Saudi Arabia, Kuwait and United Arab Emirates have been among the most substantial donors in the world relative to national economy [7]. Previous estimates of Arab official development assistance suggested that these countries provided an exceptionally large amount of aid relative to their gross national income in the last century (ranging from 8.5 to 12% in the 1970s) [8]. Since 2002, development assistance has increased both in level and as a share of gross national income as oil prices have risen and post-conflict reconstruction needs have expanded. These three countries (Saudi Arabia, Kuwait and United Arab Emirates) also have established a number of specialized financial institutions to provide development aid for Arab and Muslim countries and other developing countries [9], and gradually expanded their aid focus from infrastructure to other social sectors. According to OECD 2015 estimates, Saudi Arabia and United Arab Emirates are among the top ten providers of net official development assistance [10].

Nonetheless, little is known about how much MENA country donors are contributing to global health assistance. This is mainly due to limited and sporadic reporting of development assistance disbursements. Also part of the development assistance resources from Arab countries has traditionally been channeled through ruling families in private or through government channels that are not transparent [11], and only more recently have these MENA country donors started to publicize their aid information. This is illustrated by United Arab Emirates becoming the first ever participant of the OECD DAC in 2014 [12] and other countries stepping up engagement with the DAC and the World Bank [8]. However, these estimates can be used to assess the adequacy and quality of the available resources for global health development and to advocate for improvements for countries in need.

In this study, we addressed the gap by estimating the amount of DAH contributed by MENA country donors (specifically Saudi Arabia and Kuwait) from 2000 to 2017, disaggregating by health focus area and recipient, as well as comparing it with the amount of DAH received by MENA countries to highlight the financial flow in the region.

## Methods

### Overview

We tracked DAH provided and received by the MENA region from 2000 to 2017 using methods developed by IHME [1]. We defined DAH as the in-kind and financial resources transferred to low- and middle-income countries with the primary goal to maintain or improve health. We captured flow of resource for DAH from originating sources, through disbursing agencies (channel), and to recipient and health focus area.

Our definition of the MENA region is based on the Global Burden of Disease super-region where countries were divided to seven super-regions. Global Burden of Disease super-region categorizes countries based on geographic closeness and epidemiological similarity [13]. Countries and territories included for analysis were Afghanistan, Algeria, Bahrain, Egypt, Iran, Iraq, Jordan, Kuwait, Lebanon, Libya, Morocco, Oman, Palestine, Qatar, Saudi Arabia, Sudan, Syria, Tunisia, Turkey, United Arab Emirates, and Yemen.

To generate DAH provided by the MENA region, we leveraged official development assistance data reported to OECD DAC, project-level and aggregate-level government agency budgets, annual reports, as well as financial statements from key international development agencies. After initial literature review, we restricted the bilateral aid sources to Saudi Arabia, Kuwait and United Arab Emirates, as these three individual donors provided the majority – over 90% - of official development assistance from MENA region [8]. We also included aid contributions to multilateral entities such as the United Nations agencies from these three donors and other MENA countries (Afghanistan, Algeria, Bahrain, Egypt, Iran, Iraq, Jordan, Lebanon, Libya, Morocco, Oman, Palestine, Qatar, Sudan, Syria, Tunisia and Turkey). Additionally, we captured DAH disbursed through the Islamic Development Bank, selected among various regional financial institutions due to data availability.

We estimated DAH provided to all MENA countries using data from IHME’s Financing Global Health 2018 Development Assistance for Health database (which includes project-level disbursement information of DAH from key development agencies) [1], in addition to data from the above-mentioned donors to capture the internal transfer of resources within the MENA region. Our channels of DAH included bilateral aid agencies, development banks, United Nations agencies, public-private partnerships, non-governmental organizations and foundations. A step-by-step method of how we produced the estimates can be found in the Additional file 1.

As the delivery and implementation of loans and grants requires that donor/grant making institutions incur some additional cost in terms of program management and staffing, we also calculated administrative expenses for Saudi Arabia, Kuwait and United Arab Emirates. We defined administrative expenses as the costs associated with administering grants and loans, which includes costs related to staffing and program management. Due to the lack of data, we used the average administrative cost ratio of selected development assistance agencies with more available information (from the United States, United Kingdom, Japan, Sweden and Norway) as a proxy for all the bilateral agencies, which was 12.8% [1].

### Estimating DAH disbursed through Saudi Arabia’s bilateral agencies

We generated the estimate of bilateral DAH contributions from Saudi Arabia through aggregating health-related concessional loans disbursed through the Saudi Fund for Development (SFD) and grants disbursed by the Saudi Arabia’s government agencies [14]. For Saudi Fund for Development concessional loans, we extracted 2002–2017 project-level commitment data from the Fund’s annual reports and websites [15] and calculated the health proportion of total loans. Since we only have project-level commitment in the Saudi Fund for Development reports, we included total official development assistance disbursement data using data from the 2000–2014 DAC table (total flows by type by DAC donor) extracted from OECD database (we did not use the 2015–2017 data points due to the note which said “the 2015, 2016 and 2017 activity-level data presented for Saudi Arabia are incomplete”) [16]. We used the disbursement data to rescale project-level commitment data, to reflect the actual disbursement to commitment difference of each health concessional loan. For each individual loan, annual disbursement was estimated by dividing the total disbursement by project length, using project-level information from the annual reports and websites of all projects. For projects without a closing date, estimates were based on the average project length. We predicted 2015–2017 data using 3-year weighted average ratio of commitment and disbursement data.

For the grant-disbursing agencies, we conducted a literature and data search. we found a number of agencies that work on health and mostly humanitarian activities [7]. These agencies included the King Abdullah International Foundation for Humanitarian Activities, King Salman Humanitarian Aid and Relief Center, Saudi Campaigns, Saudi Red Crescent Authority and other governmental entities [16]. These agencies do not have project-level databases and mostly do not publish public annual reports, and all of their DAH activities reported to the OECD creditor reporting system (CRS) database are health-related humanitarian aid, thus we extracted their 2000–2017 project-level information from the United Nations Office for the Coordination of Humanitarian Affairs (UNOCHA) Financial Tracking Service website, which has a longer time-series and more detailed project-level information [17]. We included only health projects that were marked as “paid contribution” and excluded “pledged” or “committed” contributions. Since UNOCHA only captures the humanitarian projects, our estimate of Saudi Arabia’s DAH is a very modest estimate. We aggregated the loans and grants to obtain our Saudi Arabia bilateral DAH.

### Estimating DAH disbursed through Kuwait’s bilateral agencies

The Kuwait Fund for Arab Economic Development is the only aid-disbursing agency in Kuwait, providing concessional loans and also administering government grants [18]. We extracted 2000–2017 project-level commitment data from the Fund’s annual report and website [19], and similarly calculated the health proportion of concessional loans, adjusted the commitment to disbursement amount using 2000–2017 DAC table (total flows by type by DAC donor) extracted from OECD database [16]. For each individual concessional loan, annual disbursement was estimated by dividing the total disbursement by project length, using project-level information from the annual reports and websites of all projects. For projects without a closing date, estimates were based on the average project length. All grants projects did not have a reported start and closing date so we assumed that the projects were disbursed in the year that they were signed since these grants were usually small and were mostly feasibility or pilot grants.

### Estimating DAH disbursed through United Arab Emirates’ bilateral agencies

United Arab Emirates (UAE) became an OECD DAC participant country in 2014 and we extracted UAE’s bilateral DAH contribution from IHME’s Financing Global Health 2018 Development Assistance for Health database [1].

### Estimating DAH disbursed through multilateral organizations and public-private partnerships

We used data from IHME’s Financing Global Health 2018 Development Assistance for Health database to estimate DAH contributions from the MENA country donors to multilateral aid agencies. The multilateral aid agencies of interest include UN agencies (the World Health Organization, the United Nations Population Fund (UNFPA), the Joint United Nations Programme on HIV/AIDS (UNAIDS), the United Nations Children’s Fund (UNICEF)), the World Bank, the Global Fund, regional development banks including the African Development Bank and the Islamic Development bank, as well as non-governmental organizations.

Among various regional financial institutions that potentially work on health sector, we only included the Islamic Development Bank (IsDB). We excluded the rest of multilateral agencies (Arab Fund for Economic and Social Development, Arab Bank for Economic Development in Africa, OPEC fund for International Development, the Arab Gulf Program for Development) due to their relatively small size in volume of DAH disbursed and lack of adequate project-level information for 2000–2017. Our estimates of IsDB was also restricted to the Ordinary Capital Resources (OCR, concessional loans with 15–25 years’ maturity and 3–7 years’ grace period, service fee up to 1.5%) [20] and did not include the Special account Waqf Fund or Islamic Solidarity Fund for Development due to data availability. We extracted project-level commitment data for IsDB from the CRS database and adjusted the commitment to disbursement amount using total gross disbursement data extracted from OECD DAC table (official development assistance disbursements by donor) [16]. We also calculated administrative expenses using IsDB year-specific administrative cost ratio based upon information extracted from the annual reports [21]. Reasons for exclusion of the above-mentioned entities and detailed description of the original methodology used to obtain the estimates can be found in the Additional file 1.

### Estimating the health focus areas and recipients of DAH from MENA

For each individual channel as described above, we also included an analysis of the composition of health funding by recipient country and health focus area. We conducted a keyword search on each individual project description to disaggregate the health focus area (HIV/AIDS, tuberculosis, malaria, reproductive and maternal health, newborn and child health, other infectious diseases, non-communicable diseases, health system strengthening/sector-wide approaches (SWAps)).

### Comparing DAH provided and received by MENA region

We compared DAH contributions from the MENA region and DAH provided to the MENA region using data extracted from the Financing Global Health 2018 database. We calculated DAH provided and received by each individual country in 2017, annualized rate of change from 2010 to 2017, as well as the three largest disbursing agencies of DAH provided or received in 2017.

Finally, we compared DAH provided by the three largest donor countries in the MENA region (United Arab Emirates, Kuwait and Saudi Arabia) and their relative gross domestic products and government spending to examine the level of DAH in relative to their national economy and general government spending. All analysis was completed using Stata version 13. We reported DAH contributions in constant 2018 US dollars.

## Results

**Fig. 2:**
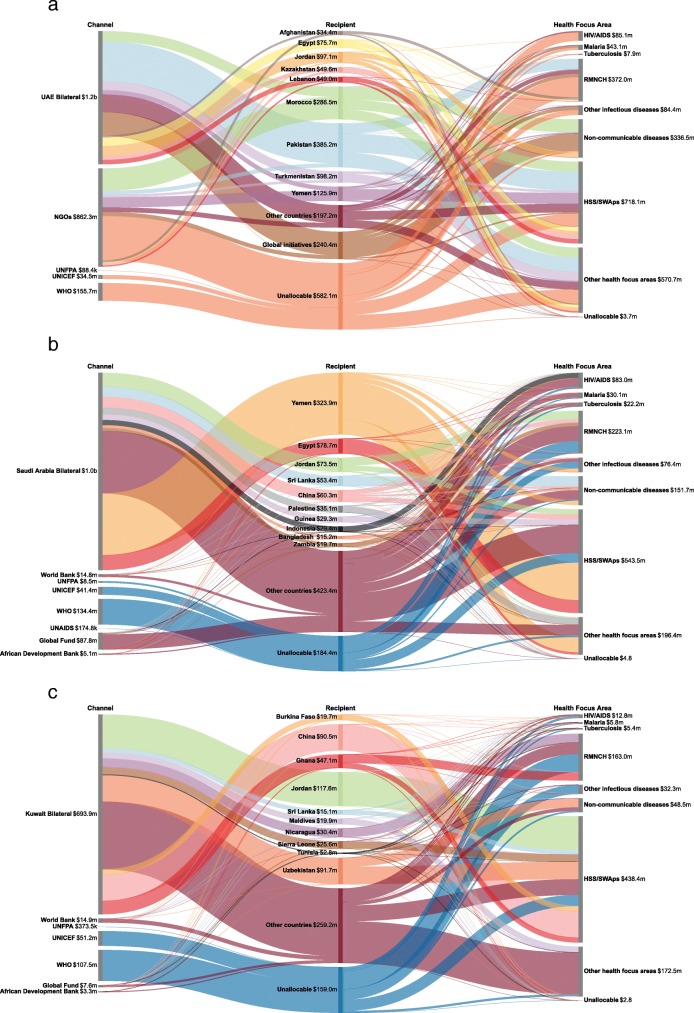
Development assistance for health contributed by **a** United Arab Emirates, **b** Saudi Arabia and **c** Kuwait by channel, recipient and health focus area, 2000–2017. Note: UAE: United Arab Emirates; NGOs: non-governmental organizations; UNICEF: United Nations Children’s Fund; UNFPA: United Nations Population Fund; WHO: World Health Organization; UNAIDS: Joint United Nations Programme on HIV/AIDS; RMNCH: reproductive, maternal, newborn and child health; HSS/SWAps: health system strengthening/sector-wide approaches

Figure 2 reports the flow of funds from the three largest MENA donors (United Arab Emirates, Saudi Arabia and Kuwait) through the various disbursing agencies, their recipients and health focus areas from 2000 through 2017. Over the past 18 years, United Arab Emirates, Saudi Arabia and Kuwait provided a total of $2.2 billion, $1.3 billion and $0.9 billion respectively. United Arab Emirates channeled $1.2 billion (52.6%) of their aid through its own bilateral agencies, $862.3 million (38.8%) through non-governmental organizations, and the rest through United Nations agencies, including WHO ($155.7 million, 7.0%), UNICEF ($34.5 million, 1.6%) and UNFPA ($0.1 million, 0.003%). Pakistan ($385.2 million, 17.3%), Morocco ($286.5 million, 12.9%) and global initiatives ($240.4 million, 10.8%) were largest recipients of DAH from United Arab Emirates, while health systems strengthening/SWAps ($718.1 million, 32.3%), reproductive, maternal, newborn and child health ($372.0 million, 16.7%) and non-communicable diseases ($336.5 million, 15.1%) were priority health focus areas for United Arab Emirates. Saudi Arabia channeled 78.0% of its DAH through its bilateral agencies, while among multilateral agencies, WHO ($134.4 million, 10.1%) and the Global Fund ($87.8 million, 6.6%) were largest channels by DAH provided. Yemen ($323.9 million, 24.4%) was the largest recipient of Saudi Arabia’s DAH, and health systems strengthening/SWAps ($543.5 million, 41.0%) was the largest health focus area of its DAH. While DAH from Kuwait was smaller in total amount, Kuwait bilateral agencies ($693.9 million, 79.0%), the World Health Organization ($107.5 million, 12.2%) and UNICEF ($51.2 million, 5.8%) channeled the majority of its DAH. Jordan ($117.6 million, 13.4%), China ($90.5million, 10.3%) and Uzbekistan ($91.7million, 10.4%) were three largest recipients of Kuwait DAH, and similarly health systems strengthening/SWAps ($438.4million, 49.9%) was the largest health focus area of its DAH.

**Fig. 3:**
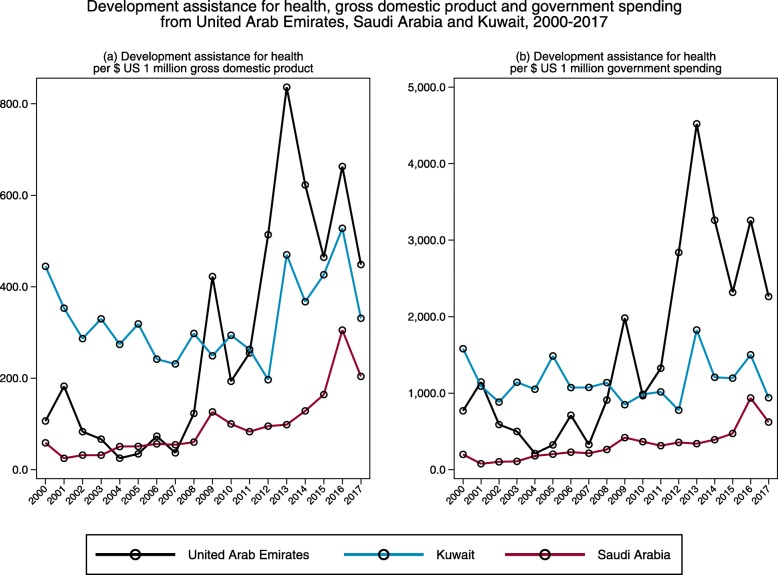
Development assistance for health per **a** gross domestic product and **b** government spending from United Arab Emirates, Saudi Arabia and Kuwait, 2000–2017

Figure 3 compares estimates of DAH from the three largest MENA country donors (United Arab Emirates, Kuwait and Saudi Arabia) with their respective gross domestic product and government spending over time. DAH per gross domestic product measured the amount of DAH contributed relative to country national income, while the DAH contribution measured relative to overall government spending is a proxy for the level of generosity exhibited. While the trends fluctuate across time, United Arab Emirates and Saudi Arabia showed an increase from 2000 to 2017. Saudi Arabia’s DAH per gross domestic product and government spending indicated a modest increase and reached its peak in 2016. Kuwait’s DAH level was highest among the three countries in 2000 ($444.5 per $1 million gross domestic product and $1580.9 per $1 million government spending), reached its peak in 2013 ($469.9 per $1 million gross domestic product and $1826.2 per $1 million government spending), and was on the decrease till 2017. United Arab Emirates provided the highest level of DAH per gross domestic product ($836.3 per $1 million) and government spending ($4521.0 per $1 million) in 2013. Despite the significantly smaller level of DAH contributed in 2017, it is still noteworthy that relative to government spending, DAH contributed by United Arab Emirates ($2263.1 per 1 million government spending) was larger than many DAC donors in 2017 (for example, Netherlands, $1735.4; Canada, $1651.9; Germany, $1045.8 per 1 million government spending).

**Fig. 4:**
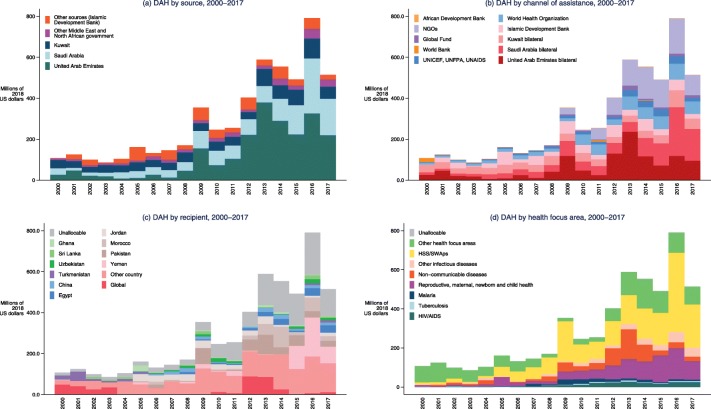
Development assistance for health from the Middle East and North Africa, 2000–2017. Note: Development assistance for health from the Middle East and North Africa by **a** source, **b** channel of disbursement, **c** recipient country and region, **d** health focus area. UNICEF: United Nations Children’s Fund; UNFPA: United Nations Population Fund; UNAIDS: Joint United Nations Programme on HIV/AIDS; HSS/SWAps: health system strengthening/sector-wide approaches

Figure 4 highlights the trends in DAH contributed by all MENA region country donors (including multilateral contribution from smaller country donors), and disaggregated by source (a), channel of assistance (b), recipient country (c) and health focus area (d). DAH contributed from MENA region increased significantly from 2000 to 2016 ($107.7 million vs. $791.2 million). In 2017, DAH contributed by MENA region was $514.8 million, down 34.9% from 2016. United Arab Emirates ($220.1 million, 42.7%), Saudi Arabia ($177.3 million, 34.4%) and Kuwait ($59.8 million, 11.6%) contributed the majority of DAH in 2017, while other MENA country government including Qatar ($14.4 million, 2.8%), Oman ($7.0 million, 1.4%) and Turkey ($7.5 million, 1.5%) also contributed a smaller proportion of DAH. 58.5% of DAH from MENA is channeled bilaterally through the three large bilateral channels, and the rest was channeled through other multilateral channel, among which the WHO and NGOs were the major disbursing agencies. The Islamic Development Bank was also one key regional channel of assistance, disbursing $22.4 million (4.4%) in 2017.

Regarding recipients of DAH from MENA, it is worth noting that 35.2% of it flows to other MENA countries e.g. Yemen and Morocco. Yemen had been a relatively small recipient before 2015, and only received 0.4% of DAH from MENA in 2013. After 2015 when the Yemen civil war exacerbated, DAH to Yemen significantly increased and in 2017 reached $81.0 million (15.7% of DAH from MENA). Egypt and Pakistan were also large recipients of DAH from MENA, receiving $36.0 million (7.0%) and $28.9 million (5.6%) in 2017. DAH from MENA has also gradually focused on non-MENA country recipients, among which Bangladesh and Mali received 4.4 and 3.7% of DAH in 2017. Across health focus area, $221.0 million or 42.9% of DAH from MENA was directed to health system strengthening and sector-wide approaches (HSS/SWAps), and $95.2 million or 18.5% was directed to reproductive, maternal, newborn and child health. HIV/AIDS, tuberculosis and malaria were not health focus areas of high priority for MENA donors, cumulatively represented $38.4 million or 7.5% of DAH in 2017.

Table 1 reports the amount of DAH received and contributed by each MENA country, and the agency each country prioritized. Out of 21 MENA countries, 15 countries were recipients of DAH, ranging from $2.2 million (Algeria) to $515.1 million (Yemen) in 2017. DAH received significantly increased from 2010 to 2017 in Libya (33.2%), Syria (20.7%) and Yemen (30.3%), where conflicts were ongoing. World Bank, European Commission and international NGOs were larger disbursing agencies for DAH received in these countries. Besides Egypt (29.96%), Jordan (19.79%) and Tunisia (35.53%), most other MENA countries did not receive a large proportion of DAH from MENA regional donors. Overall in 2017, only 10.5% of DAH received by MENA countries were from MENA donors. As for MENA countries as DAH donors, all MENA countries but Yemen contributed some DAH in 2017, with United Arab Emirates, Kuwait and Saudi Arabia being the three largest donors, followed by Qatar ($14.4 million), Turkey ($7.5 million) and Oman ($7.0 million) where we only captured multilateral contribution. The annualized rate of change in DAH provided increased significantly in Qatar (53.6%), while modest in Kuwait (4.4%). Besides bilateral channels, WHO and UNICEF were larger DAH disbursing agencies across MENA country donors.

**Table 1:** Development assistance for health in the Middle East and North Africa

Country and territory name	Total DAH received (2017)	Annualised rate of change in DAH received (2010–2017)	Largest channels by DAH received, 2017		DAH received from Middle East and North Africa region, 2017	Total DAH provided (2017)	Annualised rate of change in DAH provided, 2010–2017	Largest channels by DAH provided, 2017	
Afghanistan	234.67	−5.62%	USA Bilateral	19.84%	2.62%	1.29	18.31%	UNICEF	90.86%
			International NGO	16.55%				WHO	9.14%
			Gavi	14.31%					
Algeria	2.18	−9.44%	GFATM	32.67%	0.36%	1.05	18.11%	WHO	90.66%
			International NGO	31.91%				UNFPA	9.34%
			European Commission	10.27%					
Bahrain	0					0.23	6.93%	WHO	100.0%
Egypt	124.25	3.73%	World Bank IBRD	52.08%	28.94%	0.04	−33.25%	UNFPA	52.49%
			Saudi Arabia Bilateral	22.68%				WHO	36.42%
			United Arab Emirates Bilateral	6.26%				African Development Bank	11.10%
Iran	7.31	−6.46%	Global Fund	80.53%	0.24%	0.08	−30.72%	UNFPA	91.56%
			Japan Bilateral	9.00%				UNICEF	8.44%
			International NGO	2.64%					
Iraq	22.70	−18.71%	World Bank IBRD	48.68%	0.90%	0.68	28.12%	WHO	98.61%
			International NGO	13.05%				UNICEF	1.39%
			Global Fund	11.92%					
Jordan	85.96	1.23%	USA Bilateral	36.41%	19.79%	0.65	27.36%	UNICEF	59.54%
			World Bank IBRD	19.57%				WHO	31.96%
			International NGO	10.41%				UNFPA	8.50%
Kuwait	0					59.77	4.43%	Kuwait Bilateral	85.43%
								WHO	6.82%
								Global Fund	4.91%
Lebanon	20.41	5.63%	International NGO	49.71%	5.91%	0.02	−41.41%	UNFPA	79.80%
			Global Fund	9.29%				WHO	20.20%
			Italy Bilateral	8.36%					
Libya	8.30	33.24%	Italy Bilateral	62.69%	0.00%	0.09	−14.52%	African Development Bank	100.0%
			Netherlands Bilateral	28.25%					
			European Commission	6.63%					
Morocco	178.44	9.99%	African Development Bank	35.62%	12.78%	0.31	−16.74%	WHO	89.55%
			European Commission	22.74%				UNICEF	6.25%
			World Bank IBRD	16.86%				UNFPA	4.20%
Oman	0					7.03	42.22%	WHO	89.12%
								Gavi	8.22%
								UNICEF	2.50%
Palestine	95.24	3.29%	UK Bilateral	64.55%	0.01%	0.01	−27.35%	UNFPA	100.0%
			International NGO	8.84%					
			NGO	6.30%					
Qatar	0					14.43	53.56%	UNICEF	46.85%
								Global Fund	27.11%
								WHO	25.81%
Saudi Arabia	0					177.32	14.13%	Saudi Arabia Bilateral	87.31%
								WHO	8.21%
								Global Fund	3.31%
Sudan	113.91	−1.84%	Gavi	29.35%	0.53%	1.30	30.57%	WHO	93.07%
			Global Fund	24.36%				UNFPA	5.11%
			European Commission	14.22%				UNICEF	1.82%
Syria	75.08	20.74%	Gavi	38.35%	11.70%	0.25	−20.78%	WHO	100.0%
			Japan Bilateral	15.96%					
			European Commission	14.88%					
Tunisia	17.60	4.78%	European Commission	28.57%	35.53%	0.29	−4.95%	WHO	98.83%
			Saudi Arabia Bilateral	19.47%				UNICEF	1.17%
			Global Fund	18.67%					
Turkey	228.64	−5.60%	European Commission	76.04%	0.29%	7.49	−3.67%	WHO	84.12%
			UK Bilateral	16.75%				UNICEF	10.44%
			World Bank IBRD	5.15%				UNFPA	2.21%
United Arab Emirates	0					220.06	16.79%	United Arab Emirates Bilateral	43.23%
								International NGO	26.49%
								NGO	18.41%
Yemen	515.13	30.28%	World Bank IDA	71.56%	15.73%	0			
			Saudi Arabia Bilateral	14.80%					
			Gavi	5.02%					

DAH amounts are values expressed in millions of 2018 US dollars

*NGOs* non-governmental organizations, *UNICEF* United Nations Children’s Fund, *UNFPA* United Nations Population Fund, *WHO* World Health Organization, *UNAIDS* Joint United Nations Programme on HIV/AIDS, *IDA* International Development Association, *IBRD* International Bank for Reconstruction and Development

## Discussion

This study generated estimates of DAH contributed from the MENA region and found that in 2017, financial and in-kind resource towards global health from the MENA region reached $514.8 million, a four-fold increase from 2000. While United Arab Emirates, Saudi Arabia and Kuwait contributed as a source 88.8% of DAH from MENA in 2017, 58.5% of total DAH from MENA was channeled through their respective bilateral agencies, 12.0% through WHO and 3.3% through other United Nations agencies. Health system strengthening/SWAps was the main health focus area for DAH contributed by MENA donors.

MENA region is among the largest DAH contributing regions. Country donors like United Arab Emirates, Kuwait and Saudi Arabia are among the list of non-traditional donors, but more often labeled as re-emerging donors [22]. The Kuwait Fund for Arab Economic Development, the loan-disbursing and grant-managing agency of Kuwait was established in 1961, the same year as the United States Agency for International Development (USAID) was founded [23]; and the United Arab Emirates and Saudi Arabia founded the Abu Dhabi Fund for Development in 1971 and the Saudi Fund for Development in 1974 respectively. Historically, development aid from the Arab world has been very substantial in terms of aid as a percentage of gross national income, with a significantly higher level than the 0.7% target which was recommended by the United Nations [8]. Aid from other MENA donors have also been emerging for the last decade, including Qatar and Turkey. According to OECD estimates, Saudi Arabia and United Arab Emirates provided $6758 million and $4381 million official development assistance in 2015, ranking among top 10 country donors, while Turkey and Qatar provided an estimated $3919 and $1344 million, also among top 30 donors [10].

Our study indicated that despite these donors providing a significant amount of official development assistance in general, the health proportion of development aid is not as substantial. This is largely due to these country donors’ preferential focus on infrastructure, especially transportation, energy and other non-health related infrastructure. Unlike traditional DAC donors that provide more aid for governance and service provision, the MENA country donor agencies and regional financial institutions have been focusing their attention and activities especially to infrastructure sectors such as transportation, energy and water and sanitation [24], which is also reflected in their DAH focus area portfolio. 41.0% of Saudi Arabia,32.3% of United Arab Emirates and 49.9% of Kuwait’s DAH flows to HSS/SWAps, and most projects are hospitals and clinics constructions. Also different from other DAC donors is that, these countries do not prioritize traditional infectious diseases as their health focus areas. Cumulatively only 7.5% of DAH from MENA flows to HIV/AIDS, tuberculosis and malaria in 2017, whereas 36.4% of global DAH prioritized these health focus area [1].

Besides, large proportion of DAH from MENA country donors are channeled through bilateral basis, which coincides with their general ODA flow. Estimates from the World Bank suggested that from 1995 to 2007, 89% of total ODA offered by these countries were bilateral aid, and the rest were channeled through Arab financial institutions (4%), the World Bank (4%), UN agencies (2%) and the African Development Bank (1%) [8]. More recently, MENA country donors have started to participate in international cooperation mechanisms. In 2014, UAE became the first country outside of DAC to report to OECD CRS [12], signaling the donor country’s willingness to become more engaged with the international aid community especially the DAC instruments and standards. As for aid management, the management architecture of MENA country donors is more fragmented than DAC donors, with various governmental departments and semi-governmental departments (national Red Crescent societies), as well as regional financial institutions and “personally” established aid campaigns channeled through ruling families [7, 25]. However, it is also noteworthy that they also have formed regional coordination groups to harmonize aid policies, procurement standards and project co-financing, which differentiates these donor countries from BRICS and Southern donors as well [26].

Unlike DAC donors who experienced a dramatic increase in DAH contributions at the start of the twentieth century and plateaued after 2011 DAH from MENA country donors has always been fluctuating in amount level. A hypothesis on the relationship between development aid and oil prices suggests that peaks in Arab aid coincide with periods of increasing oil prices given the heavy dependence of donor countries’ economy on oil revenues [23–25], which may explain why DAH amount is not steady as well.

It should also be noted that DAH from MENA country donors also flows to other regions. While Arab states still prioritize aid to some of its regional counterparts, MENA country donors have increasing allocate a larger proportions of aid to countries in Sub-Saharan Africa, South and Central Asia, reflecting the newly increased diplomatic relationships with these regions due to changing global power dynamics [24, 27]. Sub-Saharan African countries received 16.5% of MENA country donors’ DAH from 2000 to 2017, the largest regional recipient after MENA. These countries are favored due to the pursuit of the Afro-Arab unity and the geopolitical links and religious solidarity between the two regions [28].

MENA region as DAH recipient received a substantial amount of DAH, due to the ongoing regional conflict. While donors from MENA region have been considered significant donors to mid-conflict and post-conflict rehabilitation activities including in Afghanistan and Yemen [7], our estimates indicated that, however, most of DAH received by the MENA region do not come from United Arab Emirates, Kuwait and Saudi Arabia. In 2017, 10.5% of DAH received by MENA countries were from MENA donors. Only Egypt (28.94%) and Tunisia (35.53%) depended on MENA donors for more than 20% of their DAH receipts. DAH channeled through World Bank and other DAC donors dwarfed the internal transfer of DAH within MENA region.

Our tracking of DAH from MENA country donors especially from Saudi Arabia and Kuwait is not without limitations, despite being the first of its kind these authors are aware of. To start with, our estimates of Saudi Arabia’s health related grants is restricted to humanitarian grants, which makes the total estimates an underestimate of total DAH contributions from Saudi Arabia. After initial literature and data search of Saudi Arabia’s grant-disbursing agencies, we found that the majority of health-related grants are all humanitarian related, thus we used UNOCHA project level data to determine the DAH grant portion. There are also a series of charitable donations by Arab rulers under “personally” established aid agencies which made the lines between official development assistance and private spending unclear [7]. We acknowledged that the list of agencies extracted from the OECD creditor reporting system is potentially incomplete, while this is by far the most comprehensive list we could get. Nonetheless, we believe that the current estimates provide the best-available evidence of DAH contribution from Saudi Arabia.

Secondly, our estimates for Kuwait and Saudi Arabia loans are based on average project length and not actual observed project disbursement amounts. We assumed that the loans projects were averagely disbursed throughout the loan start and end period, which is not usually the case. Although this will not influence our estimate for the total amount of DAH in each year, the health focus areas and recipient may slightly change across years.

Thirdly, for other MENA country donors especially Qatar and Turkey, we were only able to track their DAH contribution to multilateral institutions due to data availability. These two countries also started to disburse bilateral DAH. However, there has been limited information regarding their DAH disbursement. For example, Qatar’s 3-year available annual aid reports listed health-related disbursement varied between $110 million in 2016 to $5.3 million in 2018 [29]. Turkey’s DAH disbursement extracted from OECD dataset has been below $100 million [30]. Most importantly, both countries do not have project-level information publicly available, limiting our ability to disaggregate recipient and health focus area information, thus we did not include them for analysis.

Last but not the least, we only included Islamic Development Bank Ordinary Capital Resources (OCR) as multilateral DAH in this study. We excluded the rest of regional institutions due to their relatively small size in DAH and lack of sufficient project-level information for 2000–2018. For example, the Arab Fund only disbursed $2.18 million of health-related aid in 2017 according to the OECD Creditor Reporting System [16]; the Islamic Solidarity Fund for Development, the poverty-alleviation fund arm of the Islamic Development Bank established in 2008 only started project disbursement in 2012 and till the end of 2016, only two health projects were disbursed with a total of $18.9 million.

## Conclusion

MENA country donors especially United Arab Emirates, Saudi Arabia and Kuwait are becoming more prominent in global development including health. A total of $514.8 million DAH has been provided by MENA country donors in 2017, channeled through country bilateral agencies, WHO and other multilateral agencies. DAH from MENA is also benefiting countries outside of the MENA region and provides substantial amount towards health systems strengthening. As these donors strengthen their engagement with international development coordinating mechanisms, more detailed and comprehensive reporting of their project-level activities could be essential to global health resource tracking efforts.

## Supplementary information

**Additional file 1.** Detailed method for producing the estimates.

## Data Availability

Data generated or analysed during this study are included in this published article and its supplementary information files. The datasets used and/or analysed during the current study are also available from the corresponding author on reasonable request.

## Abbreviations

DAC: Development assistance committee
DAH: Development assistance for health
MENA: Middle East and North Africa
OECD: Organisation for Economic Co-operation and Development
UAE: United Arab Emirates
