# Supplementary material for: Development assistance for health and the Middle East and North Africa
Source: Global Health. 2020 Feb 4;16:14. doi: 10.1186/s12992-020-0545-z (PMC7001325; doi:10.1186/s12992-020-0545-z)
Supplement: Supplementary file 1 — Additional file 1. Detailed method for producing the estimates. [file 12992_2020_545_MOESM1_ESM.docx]

Development assistance for health and the Middle East and North Africa

# Supplementary method annex

[Chapter 1 Introduction 2](#_Toc10128945)

[Chapter 2 Estimating DAH from Saudi Arabia’s bilateral agencies 3](#_Toc10128946)

[Chapter 3 Estimating DAH from Kuwait’s bilateral agencies 8](#_Toc10128947)

[Chapter 4 Estimating DAH from United Arab Emirates’ bilateral agencies 11](#_Toc10128948)

[Chapter 5 Estimating DAH through multilateral organizations and public-private partnerships 12](#_Toc10128949)

[Chapter 6 Disaggregating DAH by health focus area and recipient 13](#_Toc10128950)

[Chapter 7 Comparing DAH provided and received by the Middle East and North Africa 14](#_Toc10128951)

[Reference 15](#_Toc10128952)

# Chapter 1 Introduction

I tracked development assistance for health (DAH) provided and received by the Middle East and North Africa (MENA) region from 2000-2017 using methods developed by the Institute for Health Metrics and Evaluation (IHME) (1). I defined DAH as the in-kind and financial resources transferred to low- and middle-income countries with the primary goal to maintain or improve health.

My definition of the MENA region is based on the Global Burden of Disease super-region where countries were divided to seven super-regions (2). Countries included for analysis were Afghanistan, Algeria, Bahrain, Egypt, Iran, Iraq, Jordan, Kuwait, Lebanon, Libya, Morocco, Oman, Palestine, Qatar, Saudi Arabia, Sudan, Syria, Tunisia, Turkey, United Arab Emirates, and Yemen.

To generate DAH contributed by the MENA region, I leveraged official development assistance data reported to the Development Assistance Committee (DAC) of the Organisation for Economic Co-operation and Development (OECD), project-level and aggregate-level government agency budgets, annual reports, as well as financial statements from key international development agencies. After initial literature review, we restricted our bilateral aid sources to Saudi Arabia, Kuwait and United Arab Emirates, as these three individual donors have been providing the majority – over 90% - of Arab’s official development assistance (3). We acknowledge that other countries like Qatar and Turkey have been providing DAH through their respective bilateral agencies, however, we were unable to disaggregate the aid amount by health focus areas and recipient information for Turkey due to lack of project-level information, and we were also not able to track the health proportion of Qatar’s aid. Therefore, our current estimate includes only Saudi Arabia, Kuwait and United Arab Emirates for bilateral agencies. Nonetheless, we also included aid contributions to multilateral entities such as the United Nations agencies from these three donors and other MENA countries including Qatar and Turkey. Additionally, we captured DAH disbursed through the Islamic Development Bank, among various regional financial institutions. We excluded Arab Fund for Economic and Social Development, Arab Bank for Economic Development in Africa, OPEC fund for International Development, the Arab Gulf Program for Development in our current analysis. See Chapter 5 for the reason for exclusion.

I estimated DAH received by the MENA region using data from IHME’s Financing Global Health 2018 Development Assistance for Health database (1), in addition to data from the above-mentioned donors to capture the internal transfer of resources within the MENA region. Our channels of DAH included bilateral aid agencies, development banks, United Nations agencies, public-private partnerships, non-governmental organizations and foundations.

# Chapter 2 Estimating DAH disbursed through Saudi Arabia’s bilateral agencies

I generated the estimate of Saudi Arabia’s bilateral DAH through aggregating health-related concessional loans disbursed through the Saudi Fund for Development (SFD) and grants disbursed by Saudi government agency. According to literature review, all concessional loans of Saudi Arabia were disbursed through the Saudi Fund for Development (3,6).

## Concessional Loans disbursed through Saudi Fund for Development

For Saudi Fund for Development concessional loans, I extracted 2002-2017 project-level commitment data from the Fund’s annual report and website (7).

**Table S2. Saudi Fund for Development health related concessional loans**

| Year signed | Recipient country | Project name | Cost unit | Contribution | Project signed date | Project end date |
| --- | --- | --- | --- | --- | --- | --- |
| 2017 | Tunisia | Construction and Equipment of Two Hospitals (Class B) in El Djem and Sbiba | SR million | 150 | 7/27/2017 | 12/31/2021 |
| 2017 | Kenya | Cancer Center at Kisii Hospital | SR million | 37.5 | 4/12/2017 | 4/30/2020 |
| 2017 | Swaziland | National Referral Hospital in the Kingdom of Swaziland | SR million | 37.5 | 10/14/2017 | 10/31/2019 |
| 2017 | Kyrgyzstan | Rehabilitation and Expansion of the Public Pediatric Emergency Hospital in Bishkek City | SR million | 112.5 | 5/17/2017 | 12/31/2019 |
| 2017 | Bangladesh | Construction of Burn and Pastic Surgery Unit | SR million | 112.5 | 10/8/2017 | 12/31/2021 |
| 2016 | Egypt | Development of Al Kasr Al Aini Hospital | SR million | 450 | 4/8/2016 | 1/1/2020 |
| 2016 | China | Reconstruction of Earthquake Affected Areas in Lushan District, Sichuan Province (support and provide health services to Lushan District) | SR million | 112.5 | 8/30/2016 | 12/31/2019 |
| 2015 | Sri Lanka | Epilepsy Hospital and Health Centers | SR million | 45 | 10/30/2015 | 12/31/2016 |
| 2015 | Vietnam | Extension of Hoa Binh Province General Hospital | SR million | 39 | 12/3/2015 | 12/31/2019 |
| 2014 | Guinea | Rehabilitation and Expansion of Donka Hospital in Conakry | SR million | 120 | 6/25/2014 | 12/31/2016 |
| 2014 | Uganda | Rehabilitation and Equipping of Yumbe and Kayunga Hospitals | SR million | 56.25 | 8/1/2014 | 1/31/2019 |
| 2013 | Senegal | Dalal Jamm Hospital | SR million | 34 | 5/21/2013 | 12/31/2014 |
| 2013 | Malawi | Phalombe District Hospital | SR million | 45 | 8/19/2013 | 12/31/2016 |
| 2013 | Zambia | Modernization of the University Teaching Hospital in Lusaka | SR million | 75 | 10/22/2013 | 12/31/2016 |
| 2013 | Mozambique | Construction and Equipping of Nampula General Hospital | SR million | 33.75 | 11/20/2013 | 12/31/2015 |
| 2013 | China | The Construction of the Children Hospital of Ningxia Hui Autonomous Region | SR million | 93.75 | 5/7/2013 | 6/30/2014 |
| 2013 | China | The Construction of Hanzhong Railway Central Hospital in Shaanxi Province | SR million | 93.75 | 5/7/2013 | 6/30/2014 |
| 2013 | Uzbekistan | Construction and Equipping Specialized Scientific Medical Centers with Modern Medical Equipment | SR million | 75 | 5/23/2013 | 12/31/2017 |
| 2012 | Ghana | Construction of Trauma and Acute Care Center of Korle-Bu Teaching Hospital in Accra (phase I) | SR million | 45 | 11/6/2012 | 12/31/2014 |
| 2011 | Niger | Equipping of Seven Health Centres for mother and children | SR million | 37.5 | 6/1/2011 | 12/31/2011 |
| 2011 | Burkina Faso | Construction and equipping of Manga Regional Hospital | SR million | 42 | 6/16/2011 | 6/30/2013 |
| 2011 | Vietnam | BacKan’s General Hospital and Medical Training Center | SR million | 55 | 11/1/2011 | 12/31/2014 |
| 2011 | Maldives | Seenu Hithadhoo Regional Hospital | SR million | 47 | 11/21/2011 | 12/31/2015 |
| 2010 | Benin | Construction and Equipping Project of Safy hospital | SR million | 40 | 11/9/2010 | 12/31/2013 |
| 2010 | China | Construction and Equipping of three Hospitals in Gansu Province | SR million | 75 | 4/11/2010 | 6/30/2013 |
| 2010 | Indonesia | Construction of two University Hospitals in Sebelas Maret University and Andalas University | SR million | 135 | 9/22/2010 | 12/31/2014 |
| 2010 | Cuba | Rehabilitation and Equipping of a Number of Maternity Hospitals | SR million | 75 | 4/26/2010 | 12/31/2012 |
| 2009 | Niger | Education and Health Sector Development* | SR million | 45 | 10/12/2009 | 12/31/2011 |
| 2009 | Kenya | Pediatric Emergency Center and National Burn Center at Kenyatta National Hospital | SR million | 24 | 11/14/2009 | 12/31/2012 |
| 2009 | Tajikistan | Rehabilitation and Equipping of Maternity and Child Hospital in Khujand City | SR million | 36.75 | 6/2/2009 | 12/31/2010 |
| 2009 | Bosnia & Herzegovina | Completion and Equipping of Four Hospitals | SR million | 93.75 | 7/6/2009 | 12/31/2011 |
| 2008 | Ghana | Rehabilitation and expansion of Bolgatanga Regional Hospital | SR million | 45 | 11/5/2008 | 12/31/2011 |
| 2008 | Sri Lanka | Development of Health Facilities in Colombo (additional loan) | SR million | 11 | 3/26/2008 | 12/31/2009 |
| 2008 | Sri Lanka | Epilepsy Hospital and health care centers | SR million | 75 | 3/26/2008 | 12/31/2009 |
| 2007 | Senegal | Dalal Jamm Hospital | SR million | 54.5 | 1/8/2007 | 12/31/2009 |
| 2007 | Egypt | Building & Equipping of Primary health care units | SR million | 85 | 12/16/2007 | 12/31/2011 |
| 2007 | Yemen | Faculty of Medicine and Health Sciences, University of Taiz | SR million | 45 | 11/13/2007 | 12/31/2010 |
| 2007 | Yemen | Hudaydah Central Hospital | SR million | 112.5 | 11/13/2007 | 12/31/2009 |
| 2007 | Jordan | Expansion of Al-Bashir Government Hospital | SR million | 82.5 | 12/5/2007 | 12/31/2010 |
| 2006 | Bangladesh | Medical Institutes and Specialized Hospitals | SR million | 82.5 | 8/1/2006 | 12/31/2008 |
| 2006 | Jordan | AzZarqa Government Hospital | SR million | 112.5 | 11/21/2006 | 12/31/2009 |
| 2005 | Algeria | Construction and equipping Al-Thania Hospital At Bou Merdas State. | SR million | 60 | 7/21/2005 | 12/31/2007 |
| 2005 | Morocco | Construction and equipping the university hospital at Marrakech ( additional loan). | SR million | 60 | 9/24/2005 | 12/31/2008 |
| 2005 | Morocco | Construction and equipping the university hospital at Fez ( additional loan). | SR million | 50 | 9/24/2005 | 12/31/2008 |
| 2005 | Tajikistan | Emergency care hospital in Dushanbe | SR million | 18.38 | 12/28/2005 | 12/31/2007 |
| 2004 | Rwanda | Rehabilitation and Expansion of King Faisal Hospital in Kigali | SR million | 45 | 11/1/2004 | 12/31/2007 |
| 2004 | Jordan | Expansion of Al-Bashir Government Hospital | SR million | 100 | 11/30/2004 | 12/31/2008 |
| 2003 | Turkmenistan | Constructing and Equipping of Three Diagnostic Centers | SR million | 37.5 | 5/27/2003 | 12/31/2004 |
| 2002 | Sri Lanka | Upgrading Medical Services in Colombo | SR million | 45 | 7/25/2002 | 12/31/2004 |
| 2002 | Tajikistan | Rehabilitate and Furnish Maternity Hospital at Dushanbe | SR million | 11.25 | 10/30/2002 | 6/30/2004 |

Note: For projects that covered two sectors (marked *), contribution to the health sector was estimated through diving total project contribution by two.

I calculated the health proportion of total concessional loans, which indicated how much of their total loan projects are for health. I used a constant ratio instead of using year-specific ratio due to the fact that the proportion of commitment for health not necessarily reflect the disbursement for health. I estimated that 10.6% of SFD’s concessional loans are for health. We adjusted the commitment to disbursement amount using 2000-2014 DAC1 table (total flows by type by DAC donor) extracted from OECD database (we did not use the 2015-2017 data points due to the note which said “the 2015, 2016 and 2017 activity-level data presented for Saudi Arabia are incomplete”) (4). I then generated the adjusted annual disbursement of health concessional loans from 2000-2014. I estimated 2015-2017 data using 3-year weighted average ratio of health concessional loans disbursement to health concessional loans commitment. I observed that the number in 2002 had an abnormal spike, I conducted a literature search and did not find any documentation of increase in health concessional loans in that year. The significantly large number might be due to typo in OECD DAC or increase in non-health projects, therefore I interpolated the 2002 number using 2001 and 2003 number instead.

**Table S3. Saudi Fund for Development DAH estimate**

| Year | Health concessional loan commitment (SR million) | Total commitment (SR million) | Proportion | Total concessional loan disbursed (million USD) | Health concessional loan disbursed (million USD) | DAH (million USD) |
| --- | --- | --- | --- | --- | --- | --- |
| Source | SFD report | SFD report | SFD report | OECD DAC | OECD DAC | Estimate |
| 2000 |  |  |  | 75.5 | 8.1 | 8.0 |
| 2001 |  |  |  | 59.4 | 6.3 | 6.3 |
| 2002 | 56.25 | 1271 | 0.1061651 | 1504.8 | 160.8 | 7.364# |
| 2003 | 37.5 | 839.7 | 0.1061651 | 79.3 | 8.5 | 8.4 |
| 2004 | 145 | 716.7 | 0.1061651 | 146.4 | 15.6 | 15.5 |
| 2005 | 188.38 | 802.63 | 0.1061651 | 155.2 | 16.6 | 16.5 |
| 2006 | 195 | 1029.5 | 0.1061651 | 190.3 | 20.3 | 20.2 |
| 2007 | 379.5 | 1108.25 | 0.1061651 | 200.9 | 21.5 | 21.3 |
| 2008 | 131 | 1168.13 | 0.1061651 | 223.1 | 23.8 | 23.7 |
| 2009 | 177 | 1984.5 | 0.1061651 | 529.2 | 56.5 | 56.2 |
| 2010 | 325 | 2439.25 | 0.1061651 | 320.4 | 34.2 | 34.0 |
| 2011 | 181.5 | 2392.5 | 0.1061651 | 326.0 | 34.8 | 34.6 |
| 2012 | 45 | 4111.75 | 0.1061651 | 319.1 | 34.1 | 33.9 |
| 2013 | 450.25 | 2587.75 | 0.1061651 | 318.4 | 34.0 | 33.8 |
| 2014 | 176.25 | 2571.2 | 0.1061651 | 361.4 | 38.6 | 38.4 |
| 2015 | 84 | 2454.12 | 0.1061651 |  |  | 31.9733* |
| 2016 | 562.5 | 4346.34 | 0.1061651 |  |  | 56.62592* |
| 2017 | 450 | 3936.65 | 0.1061651 |  |  | 51.28831* |

Note: The number “*” are predicted based on three year weighted average of disbursement over budget. The number “#” is interpolated using 2001 and 2003 data.

For each individual concessional loans, annual disbursement was estimated by dividing the total disbursement by project length, using project-level information from the annual reports and websites of all projects. For projects without a closing date, estimates were based on the average project length. This allowed me to average out the proportion of project disbursement in each year, to calculate the health focus area and recipient for each project in each year.

## Grants disbursed through other Saudi Arabia government agencies

In order to identify the grant-disbursing agencies, we conducted a literature and data search. I found that a number of agencies that work on health are mostly humanitarian activities (6). I was unable to generate a complete list of agencies through literature search, so based on the Creditor Reporting System (CRS) (4), I extracted the following grant-disbursing agencies for further investigation. These agencies include the King Abdullah International Foundation for Humanitarian Activities, King Salman Humanitarian Aid and Relief Center, Saudi Campaigns, Saudi Red Crescent Authority and other governmental entities. I acknowledged that the list in CRS is incomplete, while this is by far the most comprehensive list we could get. I identified seven agencies that participated in health related development work, while their project descriptions are all humanitarian-related work, including establishing mobile clinics, providing health assistance for the needy community, etc. These agencies do not have a project-level database and mostly do not have annual reports. Since the data in CRS is incomplete, therefore, we decided to use the United Nations Office for the Coordination of Humanitarian Affairs (UNOCHA) Financial Tracking Service website (8), which has a longer time-series and more detailed project-level information to track the humanitarian health-related grants.

**Table S4. List of health related grant-disbursing agencies in Saudi Arabia 2015-2017**

| Agency name | Number of projects | Aid type | Total commitment (millions of USD) | Total disbursement (millions of USD) | Any health project | Number of health projects | DAH commitment (millions of USD) | DAH disbursement (millions of USD) | Projects keywords |
| --- | --- | --- | --- | --- | --- | --- | --- | --- | --- |
| King Abdullah International Foundation for Humanitarian Activities | 4 | grant | 526 | 0 | Y | 2 | 301 | 0 | Mobile clinics, help needy communities |
| King Salman Humanitarian Aid and Relief Center | 363 | grant | 0 | 1122.544 | Y | 85 | 0 | 220.931 | Basic health care |
| Ministry of Education | 13 | grant | 0 | 66.58 | Y (Typo) | 1 | 0 | 0.05 | Scholarship |
| Other Governmental Entities | 7 | grant | 0 | 108.0104 | Y | 5 | 0 | 1.49 | Medical equipment |
| Saudi Campaigns | 28 | grant | 0 | 12.02229 | Y | 5 | 0 | 0.77 | Humanitarian aid |
| Saudi Development Fund | 109 | loan | 4057.5 | 344.345 | Y | 12 | 307 | 0 | All different kind |
| Saudi Red Crescent Authority | 6 | grant | 0 | 0.41379 | Y | 6 | 0 | 0.414 | Hospitals |
| Ministry of Foreign Affairs | 30 | grant | 0 | 30.33 | N |  |  |  | Multilateral agency share |
| Miscellaneous | 6 | grant + loan | 604.43 | 6953.067 | N |  |  |  | "Semi-aggregate data" |

We included only health projects that marked as “paid contribution” and excluded “commitment” or “pledged”. We also dropped the projects where destination organizations are United Nations agencies since we have already included them in our multilateral channels. After the data cleaning, the source organization only included the Government of Saudi Arabia, and the recipient of the projects included recipient country governments, recipient country Red Crescent societies, foundations as well as the King Salman Humanitarian Aid and Relief Center (projects carried out in Jordan, Somalia, occupied Palestinian territory). We kept the contribution amount as DAH disbursement, and ran keyword search on project description to allocate the health focus area. See chapter 6 for health focus area keyword search. Since UNOCHA only captures the humanitarian projects, our estimate of Saudi Arabia’s DAH is a very modest estimate. We finally aggregated the loans and grants to produced our Saudi Arabia bilateral DAH.

## Administrative expenses for Saudi Arabia bilateral and other bilateral agencies

As with all bilateral agencies in IHME’s Financing Global Health 2018 study, we also calculated administrative expenses for Saudi Arabia, Kuwait and United Arab Emirates. We defined administrative expenses as the costs associated with administering grants and loans. Due to the lack of agency-specific data, we used the average administrative cost ratio of selected development assistance agencies with more available administrative cost information (United States, United Kingdom, Japan, Norway and Sweden) as a proxy for all the bilateral agencies, which was 12.8% (1). We acknowledge that the administrative expense-ratio of the Arab countries might be different with other development agencies.

# Chapter 3 Estimating DAH disbursed through Kuwait’s bilateral agencies

The Kuwait Fund for Arab Economic Development is the only aid-disbursing agency in Kuwait, providing loans and also administering government grants (3). I extracted 2000-2017 project-level commitment data from the Fund’s annual report and website (9).

**Table S5. Kuwait Fund for Arab Economic Development health related concessional loans and grants**

| Year signed | Recipient country | Project name | Aid modality | Cost unit | Contribution | Project signed date | Project end date |
| --- | --- | --- | --- | --- | --- | --- | --- |
| 2018 | Mauritius | Flacq Teaching Hospital Project | Loan | KD million | 7.5 | 6/21/2018 | 12/31/2020 |
| 2017 | Tanzania | Rehab., Expansion & Equipping Hospital In Zanzibar | Loan | KD million | 4 | 9/21/2017 | 12/31/2021 |
| 2017 | China | [Development Of Hospital In Ningxia Wuzhong](https://www.kuwait-fund.org/en/web/kfund/project-information?p_p_id=projectsinformation_WAR_KFundPortletsportlet&p_p_lifecycle=0&p_p_state=normal&p_p_mode=view&p_p_col_id=column-4&p_p_col_count=1&_projectsinformation_WAR_KFundPortletsportlet_mvcPath=%2Fviews%2Fprojects-information%2FviewDetail.jsp&_projectsinformation_WAR_KFundPortletsportlet_projectId=4994&_projectsinformation_WAR_KFundPortletsportlet_backURL=%2Fen%2Fweb%2Fkfund%2Fproject-information%3Fp_p_id%3Dprojectsinformation_WAR_KFundPortletsportlet%26p_p_lifecycle%3D0%26p_p_state%3Dnormal%26p_p_mode%3Dview%26p_p_col_id%3Dcolumn-4%26p_p_col_count%3D1%26_projectsinformation_WAR_KFundPortletsportlet_radioStatus%3DAll%26_projectsinformation_WAR_KFundPortletsportlet_radioType%3DAll%26_projectsinformation_WAR_KFundPortletsportlet_radioSearchBy%3DAll%26_projectsinformation_WAR_KFundPortletsportlet_listSectors%3D7%26_projectsinformation_WAR_KFundPortletsportlet_mvcPath%3D%252Fviews%252Fprojects-information%252Fview.jsp%26_projectsinformation_WAR_KFundPortletsportlet_radioSectors%3DSector%26_projectsinformation_WAR_KFundPortletsportlet_delta%3D25%26_projectsinformation_WAR_KFundPortletsportlet_keywords%3D%26_projectsinformation_WAR_KFundPortletsportlet_advancedSearch%3Dfalse%26_projectsinformation_WAR_KFundPortletsportlet_andOperator%3Dtrue%26_projectsinformation_WAR_KFundPortletsportlet_resetCur%3Dfalse%26_projectsinformation_WAR_KFundPortletsportlet_cur%3D1) | Loan | KD million | 9 | 12/21/2017 | 12/31/2020 |
| 2017 | Tunisia | [Construction And Equipping Four Regional Hospitals](https://www.kuwait-fund.org/en/web/kfund/project-information?p_p_id=projectsinformation_WAR_KFundPortletsportlet&p_p_lifecycle=0&p_p_state=normal&p_p_mode=view&p_p_col_id=column-4&p_p_col_count=1&_projectsinformation_WAR_KFundPortletsportlet_mvcPath=%2Fviews%2Fprojects-information%2FviewDetail.jsp&_projectsinformation_WAR_KFundPortletsportlet_projectId=5370&_projectsinformation_WAR_KFundPortletsportlet_backURL=%2Fen%2Fweb%2Fkfund%2Fproject-information%3Fp_p_id%3Dprojectsinformation_WAR_KFundPortletsportlet%26p_p_lifecycle%3D0%26p_p_state%3Dnormal%26p_p_mode%3Dview%26p_p_col_id%3Dcolumn-4%26p_p_col_count%3D1%26_projectsinformation_WAR_KFundPortletsportlet_radioStatus%3DAll%26_projectsinformation_WAR_KFundPortletsportlet_radioType%3DAll%26_projectsinformation_WAR_KFundPortletsportlet_radioSearchBy%3DAll%26_projectsinformation_WAR_KFundPortletsportlet_listSectors%3D7%26_projectsinformation_WAR_KFundPortletsportlet_mvcPath%3D%252Fviews%252Fprojects-information%252Fview.jsp%26_projectsinformation_WAR_KFundPortletsportlet_radioSectors%3DSector%26_projectsinformation_WAR_KFundPortletsportlet_delta%3D25%26_projectsinformation_WAR_KFundPortletsportlet_keywords%3D%26_projectsinformation_WAR_KFundPortletsportlet_advancedSearch%3Dfalse%26_projectsinformation_WAR_KFundPortletsportlet_andOperator%3Dtrue%26_projectsinformation_WAR_KFundPortletsportlet_resetCur%3Dfalse%26_projectsinformation_WAR_KFundPortletsportlet_cur%3D1) | Loan | KD million | 24 | 11/16/2017 | 12/31/2020 |
| 2016 | Sao Tomo & Principe | [Central Hospital](https://www.kuwait-fund.org/en/web/kfund/project-information?p_p_id=projectsinformation_WAR_KFundPortletsportlet&p_p_lifecycle=0&p_p_state=normal&p_p_mode=view&p_p_col_id=column-4&p_p_col_count=1&_projectsinformation_WAR_KFundPortletsportlet_mvcPath=%2Fviews%2Fprojects-information%2FviewDetail.jsp&_projectsinformation_WAR_KFundPortletsportlet_projectId=4890&_projectsinformation_WAR_KFundPortletsportlet_backURL=%2Fen%2Fweb%2Fkfund%2Fproject-information%3Fp_p_id%3Dprojectsinformation_WAR_KFundPortletsportlet%26p_p_lifecycle%3D0%26p_p_state%3Dnormal%26p_p_mode%3Dview%26p_p_col_id%3Dcolumn-4%26p_p_col_count%3D1%26_projectsinformation_WAR_KFundPortletsportlet_radioStatus%3DAll%26_projectsinformation_WAR_KFundPortletsportlet_radioType%3DAll%26_projectsinformation_WAR_KFundPortletsportlet_radioSearchBy%3DAll%26_projectsinformation_WAR_KFundPortletsportlet_listSectors%3D7%26_projectsinformation_WAR_KFundPortletsportlet_mvcPath%3D%252Fviews%252Fprojects-information%252Fview.jsp%26_projectsinformation_WAR_KFundPortletsportlet_radioSectors%3DSector%26_projectsinformation_WAR_KFundPortletsportlet_delta%3D25%26_projectsinformation_WAR_KFundPortletsportlet_keywords%3D%26_projectsinformation_WAR_KFundPortletsportlet_advancedSearch%3Dfalse%26_projectsinformation_WAR_KFundPortletsportlet_andOperator%3Dtrue%26_projectsinformation_WAR_KFundPortletsportlet_resetCur%3Dfalse%26_projectsinformation_WAR_KFundPortletsportlet_cur%3D1) | Loan | KD million | 5 | 11/22/2016 | 12/31/2020 |
| 2016 | Sri Lanka | [Building Complex For The Faculty Of Health-care Sciences](https://www.kuwait-fund.org/en/web/kfund/project-information?p_p_id=projectsinformation_WAR_KFundPortletsportlet&p_p_lifecycle=0&p_p_state=normal&p_p_mode=view&p_p_col_id=column-4&p_p_col_count=1&_projectsinformation_WAR_KFundPortletsportlet_mvcPath=%2Fviews%2Fprojects-information%2FviewDetail.jsp&_projectsinformation_WAR_KFundPortletsportlet_projectId=4770&_projectsinformation_WAR_KFundPortletsportlet_backURL=%2Fen%2Fweb%2Fkfund%2Fproject-information%3Fp_p_id%3Dprojectsinformation_WAR_KFundPortletsportlet%26p_p_lifecycle%3D0%26p_p_state%3Dnormal%26p_p_mode%3Dview%26p_p_col_id%3Dcolumn-4%26p_p_col_count%3D1%26_projectsinformation_WAR_KFundPortletsportlet_radioStatus%3DAll%26_projectsinformation_WAR_KFundPortletsportlet_radioType%3DAll%26_projectsinformation_WAR_KFundPortletsportlet_radioSearchBy%3DAll%26_projectsinformation_WAR_KFundPortletsportlet_listSectors%3D7%26_projectsinformation_WAR_KFundPortletsportlet_mvcPath%3D%252Fviews%252Fprojects-information%252Fview.jsp%26_projectsinformation_WAR_KFundPortletsportlet_radioSectors%3DSector%26_projectsinformation_WAR_KFundPortletsportlet_delta%3D25%26_projectsinformation_WAR_KFundPortletsportlet_keywords%3D%26_projectsinformation_WAR_KFundPortletsportlet_advancedSearch%3Dfalse%26_projectsinformation_WAR_KFundPortletsportlet_andOperator%3Dtrue%26_projectsinformation_WAR_KFundPortletsportlet_resetCur%3Dfalse%26_projectsinformation_WAR_KFundPortletsportlet_cur%3D1) | Loan | KD million | 10 | 3/24/2016 | 12/31/2019 |
| 2016 | Uzbekistan | [Equipping Urological & Hemodialysis Facilities](https://www.kuwait-fund.org/en/web/kfund/project-information?p_p_id=projectsinformation_WAR_KFundPortletsportlet&p_p_lifecycle=0&p_p_state=normal&p_p_mode=view&p_p_col_id=column-4&p_p_col_count=1&_projectsinformation_WAR_KFundPortletsportlet_mvcPath=%2Fviews%2Fprojects-information%2FviewDetail.jsp&_projectsinformation_WAR_KFundPortletsportlet_projectId=5491&_projectsinformation_WAR_KFundPortletsportlet_backURL=%2Fen%2Fweb%2Fkfund%2Fproject-information%3Fp_p_id%3Dprojectsinformation_WAR_KFundPortletsportlet%26p_p_lifecycle%3D0%26p_p_state%3Dnormal%26p_p_mode%3Dview%26p_p_col_id%3Dcolumn-4%26p_p_col_count%3D1%26_projectsinformation_WAR_KFundPortletsportlet_radioStatus%3DAll%26_projectsinformation_WAR_KFundPortletsportlet_radioType%3DAll%26_projectsinformation_WAR_KFundPortletsportlet_radioSearchBy%3DAll%26_projectsinformation_WAR_KFundPortletsportlet_listSectors%3D7%26_projectsinformation_WAR_KFundPortletsportlet_mvcPath%3D%252Fviews%252Fprojects-information%252Fview.jsp%26_projectsinformation_WAR_KFundPortletsportlet_radioSectors%3DSector%26_projectsinformation_WAR_KFundPortletsportlet_delta%3D25%26_projectsinformation_WAR_KFundPortletsportlet_keywords%3D%26_projectsinformation_WAR_KFundPortletsportlet_advancedSearch%3Dfalse%26_projectsinformation_WAR_KFundPortletsportlet_andOperator%3Dtrue%26_projectsinformation_WAR_KFundPortletsportlet_resetCur%3Dfalse%26_projectsinformation_WAR_KFundPortletsportlet_cur%3D1) | Loan | KD million | 7 | 3/15/2016 | 12/31/2020 |
| 2016 | Kyrgyzstan | [Const. & Equip. Neurosurgical Center-national](https://www.kuwait-fund.org/en/web/kfund/project-information?p_p_id=projectsinformation_WAR_KFundPortletsportlet&p_p_lifecycle=0&p_p_state=normal&p_p_mode=view&p_p_col_id=column-4&p_p_col_count=1&_projectsinformation_WAR_KFundPortletsportlet_mvcPath=%2Fviews%2Fprojects-information%2FviewDetail.jsp&_projectsinformation_WAR_KFundPortletsportlet_projectId=5510&_projectsinformation_WAR_KFundPortletsportlet_backURL=%2Fen%2Fweb%2Fkfund%2Fproject-information%3Fp_p_id%3Dprojectsinformation_WAR_KFundPortletsportlet%26p_p_lifecycle%3D0%26p_p_state%3Dnormal%26p_p_mode%3Dview%26p_p_col_id%3Dcolumn-4%26p_p_col_count%3D1%26_projectsinformation_WAR_KFundPortletsportlet_radioStatus%3DAll%26_projectsinformation_WAR_KFundPortletsportlet_radioType%3DAll%26_projectsinformation_WAR_KFundPortletsportlet_radioSearchBy%3DAll%26_projectsinformation_WAR_KFundPortletsportlet_listSectors%3D7%26_projectsinformation_WAR_KFundPortletsportlet_mvcPath%3D%252Fviews%252Fprojects-information%252Fview.jsp%26_projectsinformation_WAR_KFundPortletsportlet_radioSectors%3DSector%26_projectsinformation_WAR_KFundPortletsportlet_delta%3D25%26_projectsinformation_WAR_KFundPortletsportlet_keywords%3D%26_projectsinformation_WAR_KFundPortletsportlet_advancedSearch%3Dfalse%26_projectsinformation_WAR_KFundPortletsportlet_andOperator%3Dtrue%26_projectsinformation_WAR_KFundPortletsportlet_resetCur%3Dfalse%26_projectsinformation_WAR_KFundPortletsportlet_cur%3D1) | Loan | KD million | 6 | 3/11/2016 | 12/31/2020 |
| 2016 | Vietnam | [Chu Doc General Hospital In An Giang Province](https://www.kuwait-fund.org/en/web/kfund/project-information?p_p_id=projectsinformation_WAR_KFundPortletsportlet&p_p_lifecycle=0&p_p_state=normal&p_p_mode=view&p_p_col_id=column-4&p_p_col_count=1&_projectsinformation_WAR_KFundPortletsportlet_mvcPath=%2Fviews%2Fprojects-information%2FviewDetail.jsp&_projectsinformation_WAR_KFundPortletsportlet_projectId=4772&_projectsinformation_WAR_KFundPortletsportlet_backURL=%2Fen%2Fweb%2Fkfund%2Fproject-information%3Fp_p_id%3Dprojectsinformation_WAR_KFundPortletsportlet%26p_p_lifecycle%3D0%26p_p_state%3Dnormal%26p_p_mode%3Dview%26p_p_col_id%3Dcolumn-4%26p_p_col_count%3D1%26_projectsinformation_WAR_KFundPortletsportlet_radioStatus%3DAll%26_projectsinformation_WAR_KFundPortletsportlet_radioType%3DAll%26_projectsinformation_WAR_KFundPortletsportlet_radioSearchBy%3DAll%26_projectsinformation_WAR_KFundPortletsportlet_listSectors%3D7%26_projectsinformation_WAR_KFundPortletsportlet_mvcPath%3D%252Fviews%252Fprojects-information%252Fview.jsp%26_projectsinformation_WAR_KFundPortletsportlet_radioSectors%3DSector%26_projectsinformation_WAR_KFundPortletsportlet_delta%3D25%26_projectsinformation_WAR_KFundPortletsportlet_keywords%3D%26_projectsinformation_WAR_KFundPortletsportlet_advancedSearch%3Dfalse%26_projectsinformation_WAR_KFundPortletsportlet_andOperator%3Dtrue%26_projectsinformation_WAR_KFundPortletsportlet_resetCur%3Dfalse%26_projectsinformation_WAR_KFundPortletsportlet_cur%3D1) | Loan | KD million | 3.4 | 3/11/2016 | . |
| 2016 | South Sudan | Kuwait Hospital For Women And Children | Loan | KD million | 3.5 | 12/12/2016 | . |
| 2015 | Eswatini | [The National Referral Hospital Project](https://www.kuwait-fund.org/en/web/kfund/project-information?p_p_id=projectsinformation_WAR_KFundPortletsportlet&p_p_lifecycle=0&p_p_state=normal&p_p_mode=view&p_p_col_id=column-4&p_p_col_count=1&_projectsinformation_WAR_KFundPortletsportlet_mvcPath=%2Fviews%2Fprojects-information%2FviewDetail.jsp&_projectsinformation_WAR_KFundPortletsportlet_projectId=4957&_projectsinformation_WAR_KFundPortletsportlet_backURL=%2Fen%2Fweb%2Fkfund%2Fproject-information%3Fp_p_id%3Dprojectsinformation_WAR_KFundPortletsportlet%26p_p_lifecycle%3D0%26p_p_state%3Dnormal%26p_p_mode%3Dview%26p_p_col_id%3Dcolumn-4%26p_p_col_count%3D1%26_projectsinformation_WAR_KFundPortletsportlet_radioStatus%3DAll%26_projectsinformation_WAR_KFundPortletsportlet_radioType%3DAll%26_projectsinformation_WAR_KFundPortletsportlet_radioSearchBy%3DAll%26_projectsinformation_WAR_KFundPortletsportlet_listSectors%3D7%26_projectsinformation_WAR_KFundPortletsportlet_mvcPath%3D%252Fviews%252Fprojects-information%252Fview.jsp%26_projectsinformation_WAR_KFundPortletsportlet_radioSectors%3DSector%26_projectsinformation_WAR_KFundPortletsportlet_delta%3D25%26_projectsinformation_WAR_KFundPortletsportlet_keywords%3D%26_projectsinformation_WAR_KFundPortletsportlet_advancedSearch%3Dfalse%26_projectsinformation_WAR_KFundPortletsportlet_andOperator%3Dtrue%26_projectsinformation_WAR_KFundPortletsportlet_resetCur%3Dfalse%26_projectsinformation_WAR_KFundPortletsportlet_cur%3D1) | Loan | KD million | 4.5 | 2/19/2016 | 12/31/2019 |
| 2015 | Benin | [Health Centers In Tchaourou](https://www.kuwait-fund.org/en/web/kfund/project-information?p_p_id=projectsinformation_WAR_KFundPortletsportlet&p_p_lifecycle=0&p_p_state=normal&p_p_mode=view&p_p_col_id=column-4&p_p_col_count=1&_projectsinformation_WAR_KFundPortletsportlet_mvcPath=%2Fviews%2Fprojects-information%2FviewDetail.jsp&_projectsinformation_WAR_KFundPortletsportlet_projectId=4611&_projectsinformation_WAR_KFundPortletsportlet_backURL=%2Fen%2Fweb%2Fkfund%2Fproject-information%3Fp_p_id%3Dprojectsinformation_WAR_KFundPortletsportlet%26p_p_lifecycle%3D0%26p_p_state%3Dnormal%26p_p_mode%3Dview%26p_p_col_id%3Dcolumn-4%26p_p_col_count%3D1%26_projectsinformation_WAR_KFundPortletsportlet_radioStatus%3DAll%26_projectsinformation_WAR_KFundPortletsportlet_radioType%3DAll%26_projectsinformation_WAR_KFundPortletsportlet_radioSearchBy%3DAll%26_projectsinformation_WAR_KFundPortletsportlet_listSectors%3D7%26_projectsinformation_WAR_KFundPortletsportlet_mvcPath%3D%252Fviews%252Fprojects-information%252Fview.jsp%26_projectsinformation_WAR_KFundPortletsportlet_radioSectors%3DSector%26_projectsinformation_WAR_KFundPortletsportlet_delta%3D25%26_projectsinformation_WAR_KFundPortletsportlet_keywords%3D%26_projectsinformation_WAR_KFundPortletsportlet_advancedSearch%3Dfalse%26_projectsinformation_WAR_KFundPortletsportlet_andOperator%3Dtrue%26_projectsinformation_WAR_KFundPortletsportlet_resetCur%3Dfalse%26_projectsinformation_WAR_KFundPortletsportlet_cur%3D1) | Loan | KD million | 5 | 7/31/2015 | 12/31/2019 |
| 2015 | Uzbekistan | [Equipping Clinics Of Medical Higher Schools](https://www.kuwait-fund.org/en/web/kfund/project-information?p_p_id=projectsinformation_WAR_KFundPortletsportlet&p_p_lifecycle=0&p_p_state=normal&p_p_mode=view&p_p_col_id=column-4&p_p_col_count=1&_projectsinformation_WAR_KFundPortletsportlet_mvcPath=%2Fviews%2Fprojects-information%2FviewDetail.jsp&_projectsinformation_WAR_KFundPortletsportlet_projectId=4775&_projectsinformation_WAR_KFundPortletsportlet_backURL=%2Fen%2Fweb%2Fkfund%2Fproject-information%3Fp_p_id%3Dprojectsinformation_WAR_KFundPortletsportlet%26p_p_lifecycle%3D0%26p_p_state%3Dnormal%26p_p_mode%3Dview%26p_p_col_id%3Dcolumn-4%26p_p_col_count%3D1%26_projectsinformation_WAR_KFundPortletsportlet_radioStatus%3DAll%26_projectsinformation_WAR_KFundPortletsportlet_radioType%3DAll%26_projectsinformation_WAR_KFundPortletsportlet_radioSearchBy%3DAll%26_projectsinformation_WAR_KFundPortletsportlet_listSectors%3D7%26_projectsinformation_WAR_KFundPortletsportlet_mvcPath%3D%252Fviews%252Fprojects-information%252Fview.jsp%26_projectsinformation_WAR_KFundPortletsportlet_radioSectors%3DSector%26_projectsinformation_WAR_KFundPortletsportlet_delta%3D25%26_projectsinformation_WAR_KFundPortletsportlet_keywords%3D%26_projectsinformation_WAR_KFundPortletsportlet_advancedSearch%3Dfalse%26_projectsinformation_WAR_KFundPortletsportlet_andOperator%3Dtrue%26_projectsinformation_WAR_KFundPortletsportlet_resetCur%3Dfalse%26_projectsinformation_WAR_KFundPortletsportlet_cur%3D1) | Loan | KD million | 6.5 | 3/19/2015 | 12/31/2017 |
| 2014 | Nicaragua | [Construction And Equipment Of Chinandega Hospital](https://www.kuwait-fund.org/en/web/kfund/project-information?p_p_id=projectsinformation_WAR_KFundPortletsportlet&p_p_lifecycle=0&p_p_state=normal&p_p_mode=view&p_p_col_id=column-4&p_p_col_count=1&_projectsinformation_WAR_KFundPortletsportlet_mvcPath=%2Fviews%2Fprojects-information%2FviewDetail.jsp&_projectsinformation_WAR_KFundPortletsportlet_projectId=4692&_projectsinformation_WAR_KFundPortletsportlet_backURL=%2Fen%2Fweb%2Fkfund%2Fproject-information%3Fp_p_id%3Dprojectsinformation_WAR_KFundPortletsportlet%26p_p_lifecycle%3D0%26p_p_state%3Dnormal%26p_p_mode%3Dview%26p_p_col_id%3Dcolumn-4%26p_p_col_count%3D1%26_projectsinformation_WAR_KFundPortletsportlet_radioStatus%3DAll%26_projectsinformation_WAR_KFundPortletsportlet_radioType%3DAll%26_projectsinformation_WAR_KFundPortletsportlet_radioSearchBy%3DAll%26_projectsinformation_WAR_KFundPortletsportlet_listSectors%3D7%26_projectsinformation_WAR_KFundPortletsportlet_mvcPath%3D%252Fviews%252Fprojects-information%252Fview.jsp%26_projectsinformation_WAR_KFundPortletsportlet_radioSectors%3DSector%26_projectsinformation_WAR_KFundPortletsportlet_delta%3D25%26_projectsinformation_WAR_KFundPortletsportlet_keywords%3D%26_projectsinformation_WAR_KFundPortletsportlet_advancedSearch%3Dfalse%26_projectsinformation_WAR_KFundPortletsportlet_andOperator%3Dtrue%26_projectsinformation_WAR_KFundPortletsportlet_resetCur%3Dfalse%26_projectsinformation_WAR_KFundPortletsportlet_cur%3D1) | Loan | KD million | 8.5 | 10/17/2014 | 12/31/2017 |
| 2014 | China | [Luohe Medical College Expansion](https://www.kuwait-fund.org/en/web/kfund/project-information?p_p_id=projectsinformation_WAR_KFundPortletsportlet&p_p_lifecycle=0&p_p_state=normal&p_p_mode=view&p_p_col_id=column-4&p_p_col_count=1&_projectsinformation_WAR_KFundPortletsportlet_mvcPath=%2Fviews%2Fprojects-information%2FviewDetail.jsp&_projectsinformation_WAR_KFundPortletsportlet_projectId=4410&_projectsinformation_WAR_KFundPortletsportlet_backURL=%2Fen%2Fweb%2Fkfund%2Fproject-information%3Fp_p_id%3Dprojectsinformation_WAR_KFundPortletsportlet%26p_p_lifecycle%3D0%26p_p_state%3Dnormal%26p_p_mode%3Dview%26p_p_col_id%3Dcolumn-4%26p_p_col_count%3D1%26_projectsinformation_WAR_KFundPortletsportlet_radioStatus%3DAll%26_projectsinformation_WAR_KFundPortletsportlet_radioType%3DAll%26_projectsinformation_WAR_KFundPortletsportlet_radioSearchBy%3DAll%26_projectsinformation_WAR_KFundPortletsportlet_listSectors%3D7%26_projectsinformation_WAR_KFundPortletsportlet_mvcPath%3D%252Fviews%252Fprojects-information%252Fview.jsp%26_projectsinformation_WAR_KFundPortletsportlet_radioSectors%3DSector%26_projectsinformation_WAR_KFundPortletsportlet_delta%3D25%26_projectsinformation_WAR_KFundPortletsportlet_keywords%3D%26_projectsinformation_WAR_KFundPortletsportlet_advancedSearch%3Dfalse%26_projectsinformation_WAR_KFundPortletsportlet_andOperator%3Dtrue%26_projectsinformation_WAR_KFundPortletsportlet_resetCur%3Dfalse%26_projectsinformation_WAR_KFundPortletsportlet_cur%3D1) | Loan | KD million | 9 | 6/3/2014 | 12/31/2017 |
| 2013 | Cape Verde | [Rehabilitation Of Health Services](https://www.kuwait-fund.org/en/web/kfund/project-information?p_p_id=projectsinformation_WAR_KFundPortletsportlet&p_p_lifecycle=0&p_p_state=normal&p_p_mode=view&p_p_col_id=column-4&p_p_col_count=1&_projectsinformation_WAR_KFundPortletsportlet_mvcPath=%2Fviews%2Fprojects-information%2FviewDetail.jsp&_projectsinformation_WAR_KFundPortletsportlet_projectId=4491&_projectsinformation_WAR_KFundPortletsportlet_backURL=%2Fen%2Fweb%2Fkfund%2Fproject-information%3Fp_p_id%3Dprojectsinformation_WAR_KFundPortletsportlet%26p_p_lifecycle%3D0%26p_p_state%3Dnormal%26p_p_mode%3Dview%26p_p_col_id%3Dcolumn-4%26p_p_col_count%3D1%26_projectsinformation_WAR_KFundPortletsportlet_radioStatus%3DAll%26_projectsinformation_WAR_KFundPortletsportlet_radioType%3DAll%26_projectsinformation_WAR_KFundPortletsportlet_radioSearchBy%3DAll%26_projectsinformation_WAR_KFundPortletsportlet_listSectors%3D7%26_projectsinformation_WAR_KFundPortletsportlet_mvcPath%3D%252Fviews%252Fprojects-information%252Fview.jsp%26_projectsinformation_WAR_KFundPortletsportlet_radioSectors%3DSector%26_projectsinformation_WAR_KFundPortletsportlet_delta%3D25%26_projectsinformation_WAR_KFundPortletsportlet_keywords%3D%26_projectsinformation_WAR_KFundPortletsportlet_advancedSearch%3Dfalse%26_projectsinformation_WAR_KFundPortletsportlet_andOperator%3Dtrue%26_projectsinformation_WAR_KFundPortletsportlet_resetCur%3Dfalse%26_projectsinformation_WAR_KFundPortletsportlet_cur%3D1) | Loan | KD million | 4 | 11/17/2013 | 12/31/2017 |
| 2013 | Rwanda | [Munini Hospital](https://www.kuwait-fund.org/en/web/kfund/project-information?p_p_id=projectsinformation_WAR_KFundPortletsportlet&p_p_lifecycle=0&p_p_state=normal&p_p_mode=view&p_p_col_id=column-4&p_p_col_count=1&_projectsinformation_WAR_KFundPortletsportlet_mvcPath=%2Fviews%2Fprojects-information%2FviewDetail.jsp&_projectsinformation_WAR_KFundPortletsportlet_projectId=4530&_projectsinformation_WAR_KFundPortletsportlet_backURL=%2Fen%2Fweb%2Fkfund%2Fproject-information%3Fp_p_id%3Dprojectsinformation_WAR_KFundPortletsportlet%26p_p_lifecycle%3D0%26p_p_state%3Dnormal%26p_p_mode%3Dview%26p_p_col_id%3Dcolumn-4%26p_p_col_count%3D1%26_projectsinformation_WAR_KFundPortletsportlet_radioStatus%3DAll%26_projectsinformation_WAR_KFundPortletsportlet_radioType%3DAll%26_projectsinformation_WAR_KFundPortletsportlet_radioSearchBy%3DAll%26_projectsinformation_WAR_KFundPortletsportlet_listSectors%3D7%26_projectsinformation_WAR_KFundPortletsportlet_mvcPath%3D%252Fviews%252Fprojects-information%252Fview.jsp%26_projectsinformation_WAR_KFundPortletsportlet_radioSectors%3DSector%26_projectsinformation_WAR_KFundPortletsportlet_delta%3D25%26_projectsinformation_WAR_KFundPortletsportlet_keywords%3D%26_projectsinformation_WAR_KFundPortletsportlet_advancedSearch%3Dfalse%26_projectsinformation_WAR_KFundPortletsportlet_andOperator%3Dtrue%26_projectsinformation_WAR_KFundPortletsportlet_resetCur%3Dfalse%26_projectsinformation_WAR_KFundPortletsportlet_cur%3D2) | Loan | KD million | 3.6 | 11/12/2013 | 12/31/2017 |
| 2013 | Ghana | Trauma And Acute Care Centre At Korle-bu Hospital | Loan | KD million | 4.5 | 3/7/2013 | 12/31/2020 |
| 2011 | Burkina Faso | [Manga Regional Hospital](https://www.kuwait-fund.org/en/web/kfund/project-information?p_p_id=projectsinformation_WAR_KFundPortletsportlet&p_p_lifecycle=0&p_p_state=normal&p_p_mode=view&p_p_col_id=column-4&p_p_col_count=1&_projectsinformation_WAR_KFundPortletsportlet_mvcPath=%2Fviews%2Fprojects-information%2FviewDetail.jsp&_projectsinformation_WAR_KFundPortletsportlet_projectId=4074&_projectsinformation_WAR_KFundPortletsportlet_backURL=%2Fen%2Fweb%2Fkfund%2Fproject-information%3Fp_p_id%3Dprojectsinformation_WAR_KFundPortletsportlet%26p_p_lifecycle%3D0%26p_p_state%3Dnormal%26p_p_mode%3Dview%26p_p_col_id%3Dcolumn-4%26p_p_col_count%3D1%26_projectsinformation_WAR_KFundPortletsportlet_radioStatus%3DAll%26_projectsinformation_WAR_KFundPortletsportlet_radioType%3DAll%26_projectsinformation_WAR_KFundPortletsportlet_radioSearchBy%3DAll%26_projectsinformation_WAR_KFundPortletsportlet_listSectors%3D7%26_projectsinformation_WAR_KFundPortletsportlet_mvcPath%3D%252Fviews%252Fprojects-information%252Fview.jsp%26_projectsinformation_WAR_KFundPortletsportlet_radioSectors%3DSector%26_projectsinformation_WAR_KFundPortletsportlet_delta%3D25%26_projectsinformation_WAR_KFundPortletsportlet_keywords%3D%26_projectsinformation_WAR_KFundPortletsportlet_advancedSearch%3Dfalse%26_projectsinformation_WAR_KFundPortletsportlet_andOperator%3Dtrue%26_projectsinformation_WAR_KFundPortletsportlet_resetCur%3Dfalse%26_projectsinformation_WAR_KFundPortletsportlet_cur%3D2) | Loan | KD million | 3.3 | 10/14/2011 | 12/31/2019 |
| 2011 | Sierra Leone | Health Services | Loan | KD million | 4.25 | 5/30/2011 | 12/31/2019 |
| 2009 | Kenya | [New Wajir Distric Hospital](https://www.kuwait-fund.org/en/web/kfund/project-information?p_p_id=projectsinformation_WAR_KFundPortletsportlet&p_p_lifecycle=0&p_p_state=normal&p_p_mode=view&p_p_col_id=column-4&p_p_col_count=1&_projectsinformation_WAR_KFundPortletsportlet_mvcPath=%2Fviews%2Fprojects-information%2FviewDetail.jsp&_projectsinformation_WAR_KFundPortletsportlet_projectId=3474&_projectsinformation_WAR_KFundPortletsportlet_backURL=%2Fen%2Fweb%2Fkfund%2Fproject-information%3Fp_p_id%3Dprojectsinformation_WAR_KFundPortletsportlet%26p_p_lifecycle%3D0%26p_p_state%3Dnormal%26p_p_mode%3Dview%26p_p_col_id%3Dcolumn-4%26p_p_col_count%3D1%26_projectsinformation_WAR_KFundPortletsportlet_radioStatus%3DAll%26_projectsinformation_WAR_KFundPortletsportlet_radioType%3DAll%26_projectsinformation_WAR_KFundPortletsportlet_radioSearchBy%3DAll%26_projectsinformation_WAR_KFundPortletsportlet_listSectors%3D7%26_projectsinformation_WAR_KFundPortletsportlet_mvcPath%3D%252Fviews%252Fprojects-information%252Fview.jsp%26_projectsinformation_WAR_KFundPortletsportlet_radioSectors%3DSector%26_projectsinformation_WAR_KFundPortletsportlet_delta%3D25%26_projectsinformation_WAR_KFundPortletsportlet_keywords%3D%26_projectsinformation_WAR_KFundPortletsportlet_advancedSearch%3Dfalse%26_projectsinformation_WAR_KFundPortletsportlet_andOperator%3Dtrue%26_projectsinformation_WAR_KFundPortletsportlet_resetCur%3Dfalse%26_projectsinformation_WAR_KFundPortletsportlet_cur%3D2) | Loan | KD million | 2 | 3/3/2009 | 12/31/2017 |
| 2008 | Uzbekistan | The Heart Surgery National Center | Loan | KD million | 3.373611 | 7/21/2008 | 6/30/2011 |
| 2006 | China | [Ningxia Hospital](https://www.kuwait-fund.org/en/web/kfund/project-information?p_p_id=projectsinformation_WAR_KFundPortletsportlet&p_p_lifecycle=0&p_p_state=normal&p_p_mode=view&p_p_col_id=column-4&p_p_col_count=1&_projectsinformation_WAR_KFundPortletsportlet_mvcPath=%2Fviews%2Fprojects-information%2FviewDetail.jsp&_projectsinformation_WAR_KFundPortletsportlet_projectId=3445&_projectsinformation_WAR_KFundPortletsportlet_backURL=%2Fen%2Fweb%2Fkfund%2Fproject-information%3Fp_p_id%3Dprojectsinformation_WAR_KFundPortletsportlet%26p_p_lifecycle%3D0%26p_p_state%3Dnormal%26p_p_mode%3Dview%26p_p_col_id%3Dcolumn-4%26p_p_col_count%3D1%26_projectsinformation_WAR_KFundPortletsportlet_radioStatus%3DAll%26_projectsinformation_WAR_KFundPortletsportlet_radioType%3DAll%26_projectsinformation_WAR_KFundPortletsportlet_radioSearchBy%3DAll%26_projectsinformation_WAR_KFundPortletsportlet_listSectors%3D7%26_projectsinformation_WAR_KFundPortletsportlet_mvcPath%3D%252Fviews%252Fprojects-information%252Fview.jsp%26_projectsinformation_WAR_KFundPortletsportlet_radioSectors%3DSector%26_projectsinformation_WAR_KFundPortletsportlet_delta%3D25%26_projectsinformation_WAR_KFundPortletsportlet_keywords%3D%26_projectsinformation_WAR_KFundPortletsportlet_advancedSearch%3Dfalse%26_projectsinformation_WAR_KFundPortletsportlet_andOperator%3Dtrue%26_projectsinformation_WAR_KFundPortletsportlet_resetCur%3Dfalse%26_projectsinformation_WAR_KFundPortletsportlet_cur%3D2) | Loan | KD million | 10 | 9/14/2006 | 12/31/2009 |
| 2006 | Uzbekistan | [Emergency Medical Departments](https://www.kuwait-fund.org/en/web/kfund/project-information?p_p_id=projectsinformation_WAR_KFundPortletsportlet&p_p_lifecycle=0&p_p_state=normal&p_p_mode=view&p_p_col_id=column-4&p_p_col_count=1&_projectsinformation_WAR_KFundPortletsportlet_mvcPath=%2Fviews%2Fprojects-information%2FviewDetail.jsp&_projectsinformation_WAR_KFundPortletsportlet_projectId=3101&_projectsinformation_WAR_KFundPortletsportlet_backURL=%2Fen%2Fweb%2Fkfund%2Fproject-information%3Fp_p_id%3Dprojectsinformation_WAR_KFundPortletsportlet%26p_p_lifecycle%3D0%26p_p_state%3Dnormal%26p_p_mode%3Dview%26p_p_col_id%3Dcolumn-4%26p_p_col_count%3D1%26_projectsinformation_WAR_KFundPortletsportlet_radioStatus%3DAll%26_projectsinformation_WAR_KFundPortletsportlet_radioType%3DAll%26_projectsinformation_WAR_KFundPortletsportlet_radioSearchBy%3DAll%26_projectsinformation_WAR_KFundPortletsportlet_listSectors%3D7%26_projectsinformation_WAR_KFundPortletsportlet_mvcPath%3D%252Fviews%252Fprojects-information%252Fview.jsp%26_projectsinformation_WAR_KFundPortletsportlet_radioSectors%3DSector%26_projectsinformation_WAR_KFundPortletsportlet_delta%3D25%26_projectsinformation_WAR_KFundPortletsportlet_keywords%3D%26_projectsinformation_WAR_KFundPortletsportlet_advancedSearch%3Dfalse%26_projectsinformation_WAR_KFundPortletsportlet_andOperator%3Dtrue%26_projectsinformation_WAR_KFundPortletsportlet_resetCur%3Dfalse%26_projectsinformation_WAR_KFundPortletsportlet_cur%3D2) | Loan | KD million | 6 | 6/22/2006 | 6/30/2011 |
| 2005 | Jordan | New Aqaba Hospital | Loan | KD million | 15 | 12/21/2005 | 6/30/2012 |
| 2017 | Tunisia | Feasibility study for building and equipping of the cancer disease center | Grant | KD million | 0.3 | . | . |
| 2016 | Palestine | Completion of H.H. Sheikh Sabah Alahmad Aljaber Alsabah Health Science Compound | Grant | KD million | 0.6126 | . | . |
| 2014 | Zimbabwe | Construction of 2 general hospitals in Harare city. | Grant | KD million | 0.3 | . | . |
| 2010 | Swaziland | Feasibility studies and detailed design for the national hospital in Mbabane | Grant | KD million | 0.15 | . | . |
| 2006 | Bahrain | the construction of a new health center in Abu Maher | Grant | KD million | 3 | . | . |
| 2006 | Morocco | Financing the Expansion of a Hospital and Retirement House in the City of Assila | Grant | KD million | 1 | . | . |
| 2005 | Ghana | Preparation of the Feasibility Study for the Chidren's and maternity wards in the korle bu teaching hospital | Grant | KD million | 0.17 | . | . |
| 2004 | Moldova | Feasibility study for diabetic medical center | Grant | KD million | 0.15 | . | . |
| 2004 | Maldives | Feasibility study for a new hospital in the Holow Mali Island | Grant | KD million | 0.256 | . | . |
| Projects before 2000 (not included, for reference only) | | | | | | | |
| 1999 | Lebanon | Health facilities To finance the construction of health facilities | Grant | KD million | 0.153885 | . | . |
| 1996 | Lebanon | Program for rehabilitationg medical centers. To cover part of the cost of rehabilitating the medical centers in the country. | Grant | US million | 7 | . | . |

Similar with Saudi Arabia, I calculated the health proportion of total concessional loans and grants, which indicated how much of their total projects are for health. I calculated the total across 2000 to 2017 instead using year-specific ratio due to the fact that the proportion of commitment for health not necessarily reflect the disbursement for health. I estimated that 5.2% of KFAED’s concessional loans are for health. I adjusted the commitment to disbursement amount using 2000-2017 DAC1 table extracted from OECD database (4). I then generated the adjusted annual disbursement of health concessional loans and grants from 2000-2017.

**Table S6. Kuwait Fund for Arab Economic Development DAH estimate**

| Year | Health loan and grant commitment (KD million) | Total loan and grant commitment (KD million) | Proportion | Total loan disbursed (million USD) | Health loan disbursed (million USD) | DAH (million USD) |
| --- | --- | --- | --- | --- | --- | --- |
| Source | KFAED report | KFAED report | KFAED report | OECD DAC | OECD DAC |  |
| 2000 | . | 120.2 | 0.0521 | 431.2 | 22.5 | 22.5 |
| 2001 | . | 197.7 | 0.0521 | 385.5 | 20.1 | 20.1 |
| 2002 | . | 126.7 | 0.0521 | 322.0 | 16.8 | 16.8 |
| 2003 | . | 112.7 | 0.0521 | 453.8 | 23.7 | 23.7 |
| 2004 | 0.4 | 200.4 | 0.0521 | 412.1 | 21.5 | 21.5 |
| 2005 | 15.2 | 201.3 | 0.0521 | 555.9 | 29.0 | 29.0 |
| 2006 | 17.0 | 142.9 | 0.0521 | 466.2 | 24.3 | 24.3 |
| 2007 | 0.0 | 75.1 | 0.0521 | 487.3 | 25.4 | 25.4 |
| 2008 | 3.4 | 199.1 | 0.0521 | 662.4 | 34.5 | 34.5 |
| 2009 | 2.0 | 200.3 | 0.0521 | 527.7 | 27.5 | 27.5 |
| 2010 | 0.2 | 200.2 | 0.0521 | 616.8 | 32.2 | 32.2 |
| 2011 | 7.6 | 212.4 | 0.0521 | 495.3 | 25.8 | 25.8 |
| 2012 | 12.1 | 212.0 | 0.0521 | 451.4 | 23.5 | 23.5 |
| 2013 | 17.8 | 261.0 | 0.0521 | 495.5 | 25.8 | 25.8 |
| 2014 | 16.0 | 246.7 | 0.0521 | 549.9 | 28.7 | 28.7 |
| 2015 | 35.5 | 299.5 | 0.0521 | 631.7 | 32.9 | 32.9 |
| 2016 | 37.3 | 326.5 | 0.0521 | 1354.1 | 70.6 | 70.6 |
| 2017 | 7.5 | 284.7 | 0.0521 | 848.5 | 44.2 | 44.2 |

For each individual loan and grant project, annual disbursements were estimated by dividing the total disbursement by the project length. All grants projects did not have a reported start and closing date so we assumed that the projects were disbursed in the year that they were signed since these grants were usually small and mostly feasibility or pilot grants.

# Chapter 4 Estimating DAH disbursed through United Arab Emirates’ bilateral agencies

United Arab Emirates (UAE) became an OECD DAC participant country in 2014 and we extracted UAE’s bilateral DAH contribution from reported estimates in the IHME’s Financing Global Health 2018 Development Assistance for Health database (1). In the Financing Global Health 2018 analysis, project-level disbursement data was extracted from the OECD Creditor’s Reporting System (CRS) from 2009 to 2017 as with other traditional bilateral agencies like the United States. For earlier years’ (2000-2008) project disbursement, data was provided through personal correspondence from the Ministry of Foreign Affairs (10).

Please refer to IHME’s Financing Global Health 2018 Supplementary Methods Annex at

<http://www.healthdata.org/sites/default/files/files/policy_report/FGH/2019/FGH2018_Methods-Annex.pdf>

# Chapter 5 Estimating DAH disbursed through multilateral organizations and public-private partnerships

We used data from IHME’s Financing Global Health 2018 Development Assistance for Health database to estimate DAH contributions from the MENA country donors to multilateral aid agencies (1). Our multilateral aid agencies of interest include UN agencies (the World Health Organization, the United Nations Population Fund (UNFPA), the Joint United Nations Programme on HIV/AIDS (UNAIDS), the United Nations Children’s Fund (UNICEF)), the World Bank, the Global Fund, regional development banks including the African Development Bank and the Islamic Development bank, as well as non-governmental organizations.

We also identified a number of regional organizations that participate in development assistance (3,4). After data and literature review, we excluded the majority of multilateral agencies (Arab Monetary Fund, Arab Fund for Economic and Social Development, Arab Bank for Economic Development in Africa, OPEC fund for International Development, the Arab Gulf Program for Development) due to their relative small size in DAH and lack of sufficient project-level information for 2000-2017, and only included the Islamic Development Bank Ordinary Capital Resources (OCR), which disburses concessional loans with 15-25 years’ maturity and 3-7 years’ grace period, with a service fee up to 1.5%.

**Table S7. MENA regional development agencies and reasons for exclusion**

| **Agency** | **Reason for exclusion** |
| --- | --- |
| Arab Monetary Fund | Financial institution that does not provide DAH (11) |
| Arab Fund for Economic and Social Development | Total DAH disbursement in 2017 was $2.18 million according to OECD CRS (4) |
| Arab Bank for Economic Development in Africa | Total DAH disbursement in 2014 was $ 10.98 million according to OECD CRS, and no data after 2014 (4) |
| Islamic Development Bank Special account resources WAQF fund | No project related information and not official development assistance (12) |
| Islamic Solidarity Fund for Development | Started project disbursement in 2012 and till the end of 2016, only two health projects were disbursed with a total of $18.9 million according to annual report |
| OPEC fund for International Development | Total DAH disbursement in 2017 was $5.0 million according to OECD CRS (4) |
| The Arab Gulf Program for Development | Arab countries’ contribution to the United Nations agencies (3,7), which are already captured in the IHME Financing Global Health 2018 dataset |

## Estimating DAH through the Islamic Development Bank

As mentioned above, among various regional financial institutions that potentially work on health sector, we only included the Islamic Development Bank (IsDB). Our estimates of IsDB also restricted to the Ordinary Capital Resources (OCR) and did not include the Special account Waqf Fund or Islamic Solidarity Fund for Development as mentioned in table 7. We extracted project-level commitment data for IsDB from the CRS database and adjust the commitment to disbursement amount using total gross disbursement data extracted from OECD DAC2a table (ODA disbursements (i.e. actual expenditures) by donor (donor country or multilateral organisation) to each recipient country, along with a breakdown of certain types of aid (grants, loans, technical cooperation, developmental food aid, humanitarian aid) (4).

**Table S8. Islamic Development Bank DAH estimate**

| Year | Total ODA disbursement ($US million) | Total ODA commitment ($US million) | Disbursement to commitment ratio | DAH commitment ($US million) | DAH disbursement ($US million) |
| --- | --- | --- | --- | --- | --- |
| Source | OECD DAC | OECD CRS |  | OECD CRS |  |
| 2000 | 7.1 | 206.1 | 0.0344 | 7.6 | 0.3 |
| 2001 | 133.7 | 221.4 | 0.6038 | 25.7 | 15.5 |
| 2002 | 106.2 | 262.5 | 0.4045 | 46.2 | 18.7 |
| 2003 | 26.2 | 387.0 | 0.0676 | 31.4 | 2.1 |
| 2004 | 227.0 | 262.6 | 0.8644 | 18.6 | 16.0 |
| 2005 | 225.3 | 366.6 | 0.6146 | 73.0 | 44.9 |
| 2006 | 307.4 | 464.3 | 0.6621 | 17.9 | 11.9 |
| 2007 | 271.4 | 382.4 | 0.7097 | 49.7 | 35.3 |
| 2008 | 253.4 | 394.6 | 0.6422 | 25.4 | 16.3 |
| 2009 | 484.7 | 407.5 | 1.1892 | 40.5 | 48.2 |
| 2010 | 383.9 | 370.6 | 1.0358 | 29.7 | 30.8 |
| 2011 | 378.7 | 365.3 | 1.0367 | 17.2 | 17.8 |
| 2012 | 279.6 | 453.4 | 0.6167 | 76.6 | 47.2 |
| 2013 | 236.9 | 462.5 | 0.5122 | 48.0 | 24.6 |
| 2014 | 245.9 | 327.7 | 0.7503 | 66.1 | 49.6 |
| 2015 | 194.9 | 428.8 | 0.4545 | 53.2 | 24.2 |
| 2016 | 347.5 | 440.7 | 0.7886 | 56.5 | 44.6 |
| 2017 | 339.0 | 254.2 | 1.3335 | 15.1 | 20.2 |

# Chapter 6 Disaggregating DAH by health focus area and recipient

For each individual channel as described above, we also included an analysis of the composition of health funding by health focus area. Health focus areas of interest include those used in previous research, including HIV/AIDS, malaria, tuberculosis, reproductive and maternal health, newborn and child health, other infectious diseases, non-communicable diseases, health system strengthening/sector-wide approaches, and other We conducted a keyword search for each individual project description to disaggregate the health focus area using the same methodology as in Chang et al.(52). We also disaggregated DAH channeled through each individual channel by recipient information. For the bilateral channels, recipient country information was extracted from project-level data.

For a more detailed step-by-step explanation of how health focus area are assigned, please refer to IHME’s Financing Global Health 2018 Supplementary Methods Annex at Page 49 <http://www.healthdata.org/sites/default/files/files/policy_report/FGH/2019/FGH2018_Methods-Annex.pdf>

# Chapter 7 Comparing DAH provided and received by the Middle East and North Africa

We compared DAH provided by the three largest donor countries in the MENA region (United Arab Emirates, Kuwait and Saudi Arabia) and their relative gross domestic product and government spending to analyze the relative trends and association. Data for gross domestic product and government spending were extracted from IHME’s Financing Global Health 2018 dataset (1).

Finally, we compared DAH contributions from the MENA region to DAH received in the MENA region using data extracted from the Financing Global Health 2018 database (1). We calculated DAH provided and received by each individual country in 2017, annualized rate of change from 2010-2017, as well as the three largest channels of DAH provided/received in 2017.

# Reference

1. Chang AY, Cowling K, Micah AE, Chapin A, Chen CS, Ikilezi G, et al. Past, present, and future of global health financing: a review of development assistance, government, out-of-pocket, and other private spending on health for 195 countries, 1995–2050. The Lancet [Internet]. 2019 Apr 25 [cited 2019 May 4]; Available from: http://www.sciencedirect.com/science/article/pii/S0140673619308414

2. IHME. GBD Frequently Asked Questions [Internet]. Institute for Health Metrics and Evaluation. 2014 [cited 2019 May 12]. Available from: http://www.healthdata.org/gbd/faq

3. World Bank. Arab Development Assistance [Internet]. 2010 [cited 2019 May 4]. Available from: http://siteresources.worldbank.org/INTMENA/Resources/ADAPub82410web.pdf

4. OECD. OECD Statistics [Internet]. [cited 2018 Dec 18]. Available from: https://stats.oecd.org/index.aspx?queryid=169

5. Turkish Development Assistance Reports - TİKA [Internet]. [cited 2019 May 30]. Available from: https://www.tika.gov.tr/en/publication/list/turkish_development_assistance_reports-24

6. DFID. Political Economy of Saudi Arabia’s Overseas Aid Assistance (GSDRC Helpdesk Research Report) [Internet]. GOV.UK. 2012 [cited 2019 May 4]. Available from: https://www.gov.uk/dfid-research-outputs/political-economy-of-saudi-arabia-s-overseas-aid-assistance-gsdrc-helpdesk-research-report

7. SFD. Publications - SFD [Internet]. [cited 2019 May 27]. Available from: https://www.sfd.gov.sa/en/web/guest/publications

8. UN OCHA. Financial Tracking Service [Internet]. [cited 2019 May 4]. Available from: https://fts.unocha.org/

9. Kuwait Fund Annual Report [Internet]. [cited 2019 May 27]. Available from: https://www.kuwait-fund.org/en/web/kfund/anuual-reports

10. Al Anood Al Abdool. United Arab Emirates Ministry of Foreign Affairs and International Cooperation. UAE Foreign Assistance in Health 1990-2008 through personal correspondence. 2018; published online Jan 24.

11. صندوق النقد العربي | ARAB MONETARY FUND [Internet]. [cited 2019 May 30]. Available from: https://www.amf.org.ae/en

12. The Islamic Development Bank approves US $1096 million of funding for development projects in its member countries [Internet]. [cited 2019 May 30]. Available from: https://www.isdb.org/news/the-islamic-development-bank-approves-us-1096-million-of-funding-for-development-projects-in-its-member-countries
